# Mouse Models of Polyglutamine Diseases in Therapeutic Approaches: Review and Data Table. Part II

**DOI:** 10.1007/s12035-012-8316-3

**Published:** 2012-09-04

**Authors:** Pawel M. Switonski, Wojciech J. Szlachcic, Agnieszka Gabka, Wlodzimierz J. Krzyzosiak, Maciej Figiel

**Affiliations:** Institute of Bioorganic Chemistry, Polish Academy of Sciences, Noskowskiego 12/14, 61-704 Poznan, Poland

**Keywords:** Polyglutamine, Mouse models, Therapy, Huntington disease, Spinocerebellar ataxia, DRPLA, SBMA

## Abstract

Mouse models of human diseases are created both to understand the pathogenesis of the disorders and to find successful therapies for them. This work is the second part in a series of reviews of mouse models of polyglutamine (polyQ) hereditary disorders and focuses on in vivo experimental therapeutic approaches. Like part I of the polyQ mouse model review, this work is supplemented with a table that contains data from experimental studies of therapeutic approaches in polyQ mouse models. The aim of this review was to characterize the benefits and outcomes of various therapeutic strategies in mouse models. We examine whether the therapeutic strategies are specific to a single disease or are applicable to more than one polyQ disorder in mouse models. In addition, we discuss the suitability of mouse models in therapeutic approaches. Although the majority of therapeutic studies were performed in mouse models of Huntington disease, similar strategies were also used in other disease models.

**Electronic supplementary material:**

The online version of this article (doi:10.1007/s12035-012-8316-3) contains supplementary material, which is available to authorized users.

## Introduction

Polyglutamine (polyQ) diseases are dominantly inherited disorders caused by mutations in single genes, called expansions, that result in the excessive elongation of CAG triplet tracts encoding glutamines. This type of mutation usually produces many symptoms that are primarily, but not exclusively, neurological. Currently, nine polyQ diseases have been identified, including Huntington disease (HD); spinocerebellar ataxia (SCA) types 1, 2, 3, 6, 7 and 17; dentatorubral–pallidoluysian atrophy (DRPLA); and spinal and bulbar muscular atrophy (SBMA). Although the genes where the mutation tracts are located do not belong to common gene families, the pathogenic features caused by the mutations are similar. The symptoms of these disorders include motor impairments such as dystonia and chorea in HD, ataxia in SCAs and general muscle weakness in SBMA, which often confine the patients to a wheelchair. In some cases, serious cognitive deficiencies appear at later stages of the disease [1, 2]. The most powerful tools for studying polyQ diseases are transgenic mouse models. These models are created to explore two aspects: the disease process and potential therapies. In part I, we proposed a systematic list of phenotypes that will facilitate the characterization of mouse models and the disease process. The second aspect, finding new therapies, is discussed in the present review (part II) and is very important because polyQ diseases are currently incurable.

Many excellent and useful reviews have been published on the topic of preclinical therapy for HD and other polyQ diseases [3–6]. Here, we present an overview of the therapeutic strategies that have been tested in mouse models of polyQ diseases; more importantly, we provide an Excel data table (referred to as the data table and available in the Supplementary Materials) that lists data from papers devoted to the study of polyQ mouse models and therapies. In this table, we provide data about behavioral and molecular protocols that are used for testing the therapeutic potential of the substances and strategies that are employed in mouse models of polyQ diseases. The data table, which lists nearly 250 therapeutic approaches that were carefully selected, may also serve as a basis for assessing the predictive validity (that is, a model’s suitability for preclinical therapy) of polyQ mouse models.

**Fig. 1:**
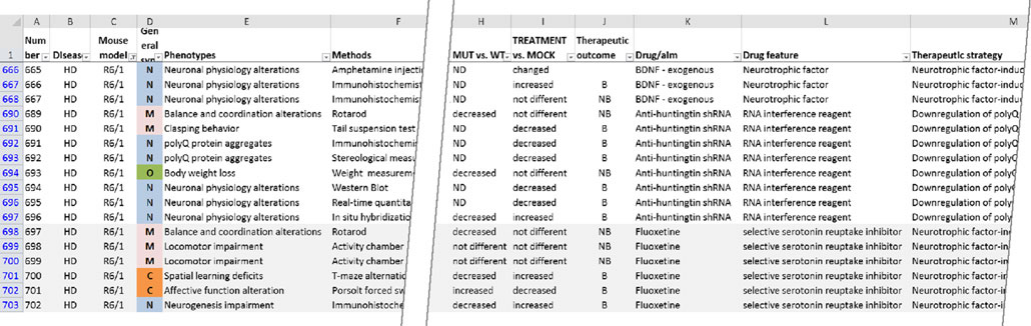
The data table is an electronic resource that provides data about the therapeutic strategies, the used behavioral and molecular protocols for testing the therapy, therapeutic substances and therapeutic outcome in mouse models. The figure demonstrates only a small fragment of the data table, and the selection of records for this figure is accidental. The full data table comprises approximately 2,000 records and 17 columns

The present work is organized into several sections. The sections “Target: Clearance Machinery” to “Other Therapeutic Strategies” contain a review of the therapeutic strategies and active substances that have been used in preclinical therapeutic trials. The following section “PolyQ Mouse Models in Experimental Therapies” discusses the polyQ mouse models that were used in therapeutic trials and the phenotypes that were used to determine therapeutic outcomes. This structure is also reflected in the data table, which contains 15 columns (Fig. 1). The first two columns list the diseases and the mouse models of the diseases that were used for the experimental therapy. A second group of columns describes the phenotypes tested, states the methods used to test the phenotypes, lists the parameters that were quantified and presents the outcome of the therapy (in the column called “Treatment vs. mock”). The third set of columns contains data about the active substances used to induce the therapeutic effect, the description of the drug target and the general therapeutic strategy. Supplemental Table 1 summarizes the content of the columns in the data table.

## Strategies and Targets of PolyQ Experimental Therapy Approaches in Mouse Models

**Fig. 2:**
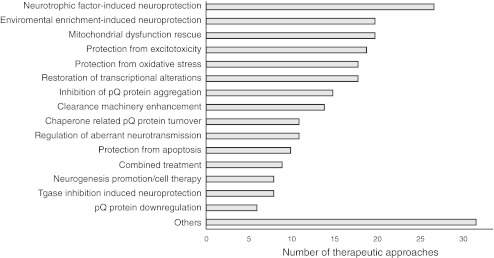
The diagram shows the most studied therapeutic strategies. The therapeutic strategies are ranked by the number of therapeutic approaches that were testing a given strategy. The data table collects the total number of 250 different therapeutic approaches. The most extensively tested strategies are related to the induction of neuroprotection (with neurotrophic factors or by exposing animals to environmental stimuli), mitochondrial dysfunction, or transcriptional deregulation. Interestingly, therapeutic approaches aimed at the specific downregulation of polyQ protein expression are rarely tested in mouse models

The pathologically elongated polyglutamine domain has a tendency to misfold and aggregate into larger structures that eventually precipitate from cytoplasmic and nucleoplasmic solutions as insoluble inclusions; however, it is still unclear whether monomers, soluble oligomers or insoluble inclusions make the greatest contribution to the overall cytotoxicity of polyglutamine repeats. Mutated polyglutamine domains interact with other cellular components and, as a result, perturb cellular homeostasis. This disruption leads to a variety of cellular dysfunctions, including transcriptional deregulation, mitochondrial dysfunction, clearance machinery impairment, increased susceptibility to excitotoxicity, inflammation and oxidative damage, and apoptosis induction [7, 8]. The polyglutamine domain alters several cellular processes, indicating that there are many potential targets for both pharmacological and non-pharmacological interventions. The validity and the potency of targeting various cellular pathways to alleviate disease phenotypes were assessed in mouse models of polyglutamine diseases using nearly 250 different therapeutic approaches that can be grouped into several different therapeutic strategies (Fig. 2, the data table, Supplementary Tables 1–8).

### Target: Clearance Machinery

#### Ubiquitin–Proteasome System and Autophagy

**Fig. 3:**
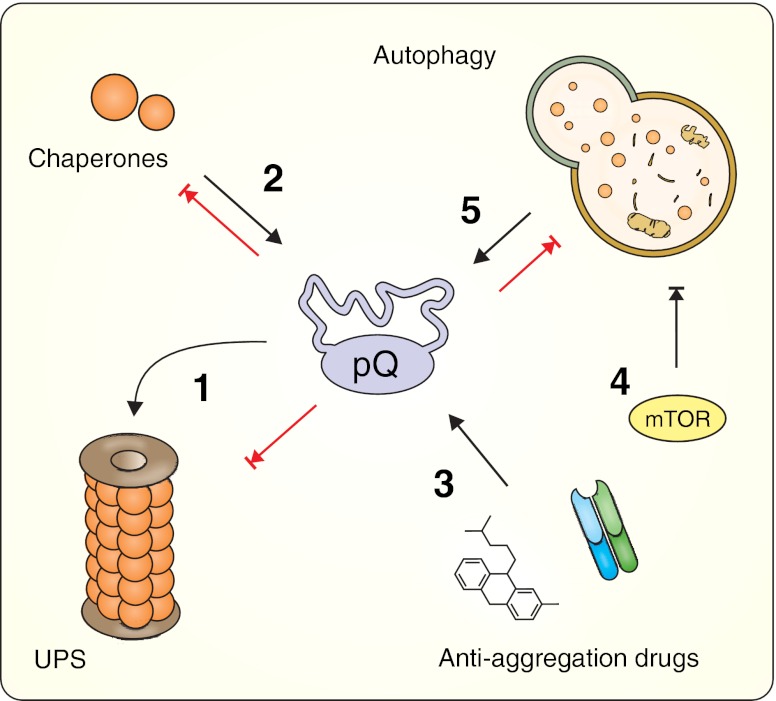
Impairment of clearance machinery in polyQ diseases. Expanded polyglutamine proteins alter the physiological functions of both the UPS and the autophagic clearance pathways, thereby perturbing cellular homeostasis. Therapeutic approaches tested in polyglutamine mouse models include facilitating UPS-mediated polyQ clearance by interfering with various steps in the UPS pathway (*1*), increasing the levels of chaperones (*2*), or administrating anti-aggregation drugs (*3*). An increase in autophagy-mediated degradation can be achieved with mTOR inhibitors (*4*) and via mTOR-independent pathways (*5*). Ubiquitin–proteasome system (*UPS*), mammalian target of rapamycin (*mTOR*)

Highly controlled and selective degradation of cellular compounds, essential for cell physiology, is executed by the ubiquitin–proteasome system (UPS) and by autophagy [9]. The UPS is a multistep pathway in which redundant or damaged proteins are tagged with ubiquitin and subsequently destroyed in the proteasome complex. In contrast, the autophagic system can eliminate both single molecules, and larger structures, such as organelles, by a process of controlled enzymatic hydrolysis of cellular compounds in the lysosome [9]. Because both autophagy and the UPS maintain protein quality by removing misfolded proteins, the accumulation of large amounts of protein containing elongated polyglutamine tracts can put stress on both pathways and, consequently, alter their physiological functions [10–13]. The explanations of the proposed mechanisms (e.g., proteasome blockade or overload, sequestering of important pathway components, and direct or indirect inhibition of the various pathway steps) are discussed elsewhere [14, 15]. The therapeutic approaches aimed at boosting cellular clearance may act in a bidirectional manner by restoring the clearance machinery function that is impaired by polyQ proteins and by accelerating the degradation of polyQ proteins. Several different strategies were used to achieve these goals in mouse model studies (Fig. 3). The therapeutic effects of autophagy upregulation were tested in the HD and SCA3 models using pharmacological inhibition of the negative regulator of autophagosome formation (mammalian target of rapamycin (mTOR)) using two derivatives of rapamycin, temsirolimus, and everolimus. Interestingly, these studies yielded contradictory results. Whereas temsirolimus accelerated mutant protein removal and improved motor performance in both 70.61 SCA3 mice and N171-82Q HD mice [16, 17], everolimus did not reduce huntingtin levels in the R6/2 mouse brain and, as a result, did not induce neuroprotection despite significant brain penetration [18].

Because mTOR regulates many cellular processes in addition to autophagy, Rose and colleagues proposed an alternative approach of autophagy stimulation to avoid the side effects caused by rapamycin and its analogs. Rilmenidine, an mTOR-independent autophagy inducer, reduced the level of the mutant huntingtin fragment and partially attenuated the disease phenotype in N171-82Q mice [19].

Enhanced proteasome degradation can be induced by perturbing various steps of the UPS pathway. Overexpression of the CRAG protein, which is an activator of promyelocytic leukemia protein-associated ubiquitin ligase, enhances the ubiquitination and proteasome clearance of mutant ataxin-3 and ultimately leads to improvements in both motor and neurological phenotypes in polyQ69 mice [20]. A similar strategy was implemented by Sobue’s group, who crossed SBMA mice with mice overexpressing CHIP, a protein with E3 ubiquitin ligase activity. Marked amelioration of the disease phenotype was correlated with the reduction of monomeric and aggregated mutant androgen receptor (AR) proteins in the spinal cords and muscles of AR-97Q mice [21]. Finally, Wong and colleagues reported that benzyl amiloride (Ben) could be used as a candidate drug in HD treatment. Ben blocks acid-sensing ion channels in R6/2 mice, which leads to an increase in UPS activity and a decrease in huntingtin aggregation [22].

#### Aggregation Process

Another strategy that takes advantage of the cellular clearing system is based on changing the polyQ protein properties that are responsible for its slower degradation rate to facilitate its clearance via the UPS pathway. One question that remains unresolved is whether nuclear inclusions play a role in the pathogenesis or are actually the product of neuroprotective mechanisms to attenuate the toxic protein fragments in the cell. Notably, in some mouse models, nuclear inclusions also appear in brain regions that are unaffected in the disease process, and often the affected brain regions contain fewer inclusions than the unaffected ones (see Part I). In contrast, the brains of mice from severely affected models, such as R6/2 and N171-82Q, often contain more inclusions than those of animals with a mild pathogenesis, such as YAC128 or HD knock-ins. Finally, the formation of inclusions in conditional mouse models can be reversed when expression of polyQ protein is switched off [23], but no studies have examined whether these aggregates are responsible for the pathology and therefore whether the reversal of the formation of inclusions would be the cause of disease amelioration.

The therapeutic approaches that target aggregation processes are based on the assumption that polyglutamine toxicity can be attributed to soluble oligomers and monomers with specific conformational structure of polyQ domains rather than to insoluble inclusions [24]. Therefore, if potential therapeutic agents could prevent mutant proteins from misfolding or oligomerizing, then toxic species would not appear, and the UPS pathway would be more effective at clearing existing polyglutamine monomers. The studies examining compounds that target aggregation processes using cellular models of polyQ diseases are not discussed here because of space constraints (for a review see [25]). In mouse model studies, this strategy was implemented by using small molecules that bind to amyloids and inhibit amyloid fibril formation or by using intrabody gene therapy or chaperone activity modulation (Fig. 3).

##### Small Molecules and Intrabodies

Small molecules that inhibit oligomerization of polyQ proteins, including chlorpromazine, minocycline, Congo red, trehalose, benzothiazoles, C2-8, and polyQ-binding peptide 1 were tested in mouse models of Huntington disease with varying success at reducing disease phenotype [26–34]. Although most of these compounds prevent inclusion formation in in vitro assays, these results have not always translated into a phenotype rescue when used in vivo to treat polyQ mice. It is possible that some compounds that inhibit inclusion body formation may prevent the soluble, toxic protein fraction from being neutralized in inclusions, inducing adverse effects of such therapeutic approach.

Intrabodies (iAbs) are engineered antibody fragments that are encoded in a vector and expressed inside cells. A number of intrabodies targeting various regions of the huntingtin protein have been developed and tested on HD mice. The mEM48-based iAb, which preferentially binds to mutant huntingtin (HTT), improves the motor performance of N171-82Q animals and reduces HTT neuropil aggregate formation; however, it is not potent enough to remove the intranuclear inclusions [35]. Patterson’s laboratory has performed extensive studies in five HD models (N171-82Q, R6/2, YAC128, BACHD, and lentiviral mouse model) with two intrabodies (VL12.3 and Happ1) and has shown strong therapeutic potential of this approach. Happ1, which recognizes polyproline and polyproline-rich domains, improves motor performance, reduces neurological and cognitive abnormalities, and prolongs average lifespan. VL12.3, an intrabody recognizing the N-terminus of HTT, has beneficial effects in lentiviral model, but does not improve phenotype in YAC128, and increases mortality in R6/2 mice. Both iAbs reduce aggregate formation in cell culture and HD mouse models [36]. Recently, Snyder-Keller and colleagues reported a reduction in the aggregate phenotype in R6/1 mouse brains, even when treatment with an intrabody that recognizes the N-terminal huntingtin region was initiated at a late stage of the disease [37]. A possible explanation of the beneficial effects of intrabodies rests in their ability to bind to the huntingtin protein, alter its conformation and therefore make the mutant huntingtin protein more accessible to the UPS system. Indeed, Wang’s intrabody seems to promote the ubiquitination and clearance of mutant huntingtin fragments [35].

##### Chaperones

Chaperones are proteins that assist in the proper folding of synthesized proteins, refold those proteins that are folded incorrectly and, together with other UPS components, recognize proteins with incorrect conformations that cannot be restored to their native states and designate them for degradation. The robust aggregation of polyQ proteins that occurs in cells of patients and animal models also indicates that chaperones cannot efficiently process the permanently misfolded polyQ stretches. Thus, increasing the levels of chaperone proteins is a reasonable approach to treating polyQ diseases. Despite numerous successes in inhibiting aggregate formation and rescuing cell death in non-mammalian and cell culture models (for example [38–40]), inducing the expression of various chaperones in polyQ mice, has had only modest effects in the amelioration of the polyglutamine-dependent disease phenotypes (Table 1). Overexpression of Hsp70, Hsp104, or BAG1 (an Hsp70 co-chaperone) or induction of chaperone expression by heat shock transcription factors does not reduce either motor or neurological abnormalities in N171-82Q or R6 HD animals, even though the aggregation processes are significantly hindered in these animals [41–45]. In contrast, overexpression of Hsp70 in SCA1 B05 and SBMA AR-97Q mice or pharmacologically increasing the levels of Hsp70, Hsp90, and Hsp105 in SBMA animals with orally administered geranylgeranylacetone improves both motor and neurological phenotypes [46–48]. Additionally, geldanamycin analogs 17-AAG and 17-DMAG, which bind directly to Hsp90, inhibit the formation of stable Hsp90/client protein complexes, and promote the formation of degradable proteasome-targeting complexes in the SBMA AR-97Q model. This strategy results in the clearing of mutant AR aggregates in both muscle tissue and in the spinal cord, which restores motor performance and prolongs the shortened lifespan [49, 50]. At present, it is not clear why the chaperone strategy works in SCA1 and SBMA models and not in HD models. First, the therapeutic effects of Hsps may depend on the expression patterns and the levels of both the chaperones and the transgenic polyQ protein. Second, because chaperones form a complex network of mutual interactions and need specific partners to function properly, increasing the level of only one Hsp protein may not be sufficient to obtain enhanced polyQ turnover in different cell types. Finally, the cause may lie within the polyQ protein itself. Induced chaperone activity was ineffective in studies that were performed using the HD mouse models with artificially truncated huntingtin fragments. Such proteins may misfold and aggregate very aggressively; consequently, chaperones may not be able to overcome this effect even when they are expressed at relatively high levels. Additional studies are needed to clarify whether enhanced chaperone activity may be beneficial in treating Huntington disease, especially in the full-length HD models.

**Table 1:** Chaperone-related therapeutic approaches in mouse models of polyQ diseases

Drug	Route/dose	Model		Therapeutic outcomes	Reference
17-AAG	Intraperitoneal (7.5 or 75 mg/kg/week)	AR-97Q (SBMA)	✓	Improved motor phenotype (rotarod, cage activity, gait pattern); alleviated aggregate formation and nuclear localization of mutant AR; reduced muscle atrophy; decreased body weight loss rate; prolonged life span	Waza et al. 2005 [49]
17-DMAG	Oral (3 or 30 mg/kg/week)	AR-97Q (SBMA)	✓	Improved motor phenotype (rotarod, cage activity, gait pattern); alleviated aggregate formation and nuclear localization of mutant AR; reduced muscle atrophy; decreased body weight loss rate; prolonged life span	Tokui et al. 2009 [50]
BAG1	Overexpression	N171-82Q (HD)	✓	Improved rotarod phenotype (only in males)	Orr et al. 2008 [43]
			×	No change in aggregate formation, body weight loss rate, life span, and clasping phenotype	
GGA	Oral (~600 and 1,200 mg/kg/day)	AR-97Q (SBMA)	✓	Improved motor phenotype (rotarod, cage activity, gait pattern); alleviated aggregate formation and nuclear localization of mutant AR; reduced muscle atrophy; decreased body weight loss rate; prolonged life span	Katsuno et al. 2005 [48]
HSF1	Overexpression	R6/2 (HD)	✓	Reduced muscular atrophy and muscular inclusions; prolonged life span	Fujimoto et al. 2005 [45]
			×	No change in clasping phenotype and body weight loss rate; no reduction in brain atrophy and neuronal inclusion formation	
hsp104	Overexpression	N171-82Q (HD)	✓	Reduced number of cortical aggregates; prolonged life span	Vacher et al. 2005 [42]
			×	No change in rotarod and grip strength performance; no change in body weight loss rate	
Hsp70	Overexpression (5- to 10-fold of endogenous level)	AR-97Q (SBMA)	✓	Improved motor phenotype (rotarod, cage activity, gait pattern); alleviated aggregate formation and nuclear localization of mutant AR; decreased body weight loss rate; prolonged life span	Adachi et al. 2003 [47]
Hsp70	Overexpression (~10- to 20-fold of endogenous level)	B05 (SCA1)	✓	Improved rotarod phenotype; improved Purkinje cell morphology	Cummings et al. 2001 [46]
			×	No change in NII formation	
Hsp70	Overexpression (5- to 15-fold of endogenous level)	R6/2 (HD)	✓	Decreased body weight loss rate	Hansson et al. 2003 [41]
			×	No change in clasping behavior; no reduction in brain atrophy and neuronal abnormal morphology; no change in NII formation and life span	
Hsp70	Overexpression	R6/2 (HD)	✓	Delayed aggregate formation in hippocampal slice culture	Hay et al. 2004 [44]
			×	No change in rotarod and grip strength performance; increased body weight loss rate	
HSP70/HDJ2	Overexpression (5- to 10-fold of endogenous level)	90Q R7E (SCA7)	×	No change in rod photoreceptor functions, no morphological changes of retinal layers, and no change in NII formation	Helmlinger et al. 2004 [392]
HSJ1a	Overexpression	R6/2 (HD)	✓	Reduced nuclear aggregate load; increased levels of soluble huntingtin; improved rotarod performance and forelimb grip strength; improved exploratory activity; increased BDNF level	Labbadia et al. 2012 [393]
			×	No change in body and brain weight loss rate	

### Target: PolyQ Protein Expression

Selective and permanent elimination of mutations from the genome would effectively cure polyQ patients; however, DNA editing technologies, such as homologous recombination or the use of zinc finger nucleases or TALENs, are difficult to translate to in vivo systems as therapeutic tools [51]. An alternative approach that can be considered etiological or preventive is to target the messenger RNA, thereby repressing the formation of the toxic polyQ protein. Davidson’s group has used RNAi in both SCA1 and HD mice. By constructing AAV vectors that can produce siRNAs inside neurons, her group has achieved potent long-term silencing of polyQ transgenes. Injection of viral particles directly into the cerebellum and striatum of B05 and N171-82Q animals, respectively, results in a significant improvement in the disease phenotype in terms of both motor and neurological impairment [52, 53]. Similar results were obtained in R6 models by using vectorized shRNA and naked siRNA against human huntingtin [54, 55].

Transgenic mice are good models that have allowed us to study the selective silencing of mutant genes without altering the expressions of the endogenous mouse counterparts [53–56], but naturally, such allele sets do not exist in the patient population. To study more natural conditions, RNAi reagents targeting sequences present in both transgenes and endogenes were used to achieve nonselective silencing. It was expected that partial elimination of wild-type allele expression would be a minimally harmful compromise for the effective removal of the polyQ protein. Interestingly, significant knockdown of endogenous huntingtin in the striatum of N171-82Q is well-tolerated even after 4 months despite the significant involvement of this protein in various cellular processes [57]. Similar results were also observed in lentiviral rat and mouse models of Huntington disease [58]. In addition, ataxin-3 knockout mice do not show any signs of gross pathology, indirectly indicating that the nonselective approach may be relatively safe [59, 60]. However, prolonging the wild-type allele silencing to years or decades in patients may lead to the gradual accumulation of undesirable effects, and eventually, such a strategy could prove to be more harmful than beneficial. To overcome this potential danger, allele-specific reagents that distinguish between the mutant and normal transcripts could be used; however, their effectiveness has only been shown in cellular and lentiviral rat models. Such allele specificity may be obtained using reagents that target SNP sites [61–63] or act through miRNA-like mechanisms that can distinguish between alleles by targeting the different lengths of the CAG repeat region in the normal and mutant transcripts [64–66].

The therapeutic strategy of targeting polyQ mRNA with RNA interference decreases the expression of mutant protein and transcripts containing elongated CAG tracts. Silencing of both components in the cell may provide additional therapeutic benefits because recently, RNA gain-of-function by transcripts harboring expanded CAG repeats has been increasingly recognized as a pathogenic factor in polyQ diseases [67, 68].

### Target: Degenerating Neurons—Neuroprotection/Neuromodulation

**Fig. 4:**
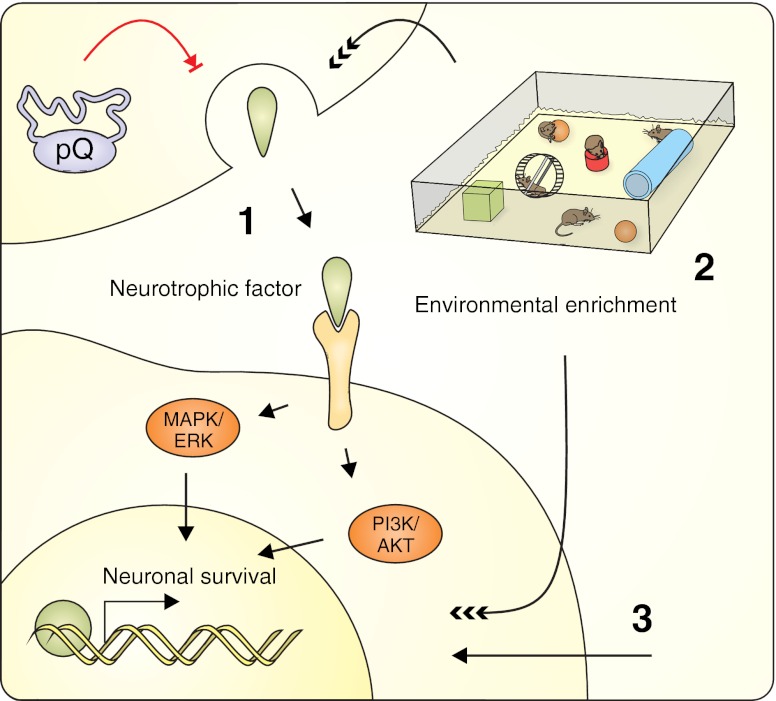
Neuroprotective and neuromodulatory strategies targeting the degenerating neurons in polyQ diseases. These experimental therapeutic strategies prevent neuronal death by supporting overall health and promoting survival. The therapy can be implemented in the following ways: by administering or inducing the expression of neurotrophic factors that promote neuronal survival (*1*); by exposing the animals to an enriched environment that results in the upregulation of endogenous neurotrophic factors and genes involved in synaptic plasticity, growth, and neurogenesis (*2*); or by other neuromodulation-related therapeutic strategies (e.g., the regulation of neurotransmitter activity) that also lead to the induction of neuroprotection (*3*). See the text for a detailed description

The most striking aspect of polyglutamine diseases is the progressive morphological and physiological degeneration, followed by the death of specific neuronal subpopulations. Several experimental therapeutic strategies have been designed to prevent neuronal death by strengthening the overall health of neurons and promoting their survival despite the neurotoxicity of the mutant polyQ protein (Fig. 4).

#### Neurotrophic Factors

Neurotrophic factors are naturally occurring signaling proteins that are essential for nervous system development and promote neuronal growth, differentiation, and the formation of neuronal connections [69]. They also play important roles in the adult brain and peripheral nervous system, where they are responsible for maintaining proper neuronal phenotypes and functions and supporting neuronal survival [70, 71]. Additionally, neurotrophic factors are involved in neuronal protection and regeneration in several neurodegenerative diseases and following neurotraumatic injuries [72–75]. The neuroprotective properties of neurotrophic factors make them attractive candidates for preventing the damage caused by mutant polyQ proteins (Table 2).

**Table 2:** Neurotrophic factor-related therapeutic approaches in mouse models of polyQ diseases

Drug	Route/dose	Model		Therapeutic outcomes	Reference
BDNF	Osmotic pump (4.5 μg/day)	R6/1 (HD)	✓	Enhanced number of encephalin + neurons	Canals et al. 2004 [80]
			×	No change in number of substance P + neurons	
BDNF	Overexpression (3- fold of endogenous level)	R6/1 (HD)	✓	Improved rotarod phenotype; decreased body weight loss rate (females); increased brain weight; normalized cortical and striatal volumes; reduced aggregates formation	Gharami et al. 2008 [86]
			×	No change in ventricle size	
BDNF	Intrastriatal injection of 6 × 10^5^ MSC cells overexpressing BDNF	YAC128 (HD)	✓	Improved rotarod and clasping behavior; reduced neuronal loss within the striatum	Dey et al. 2010 [91]
BDNF	Overexpression (2-3-fold of endogenous level)	YAC128 (HD)	✓	Improved gait pattern, rotarod, and beam walk phenotype; reversed cognitive deficits; reduced brain atrophy and loss of striatal neurons; normalized spine morphology and expression of the striatal dopamine receptor D2 and enkephalin	Xie et al. 2010 [87]
			×	No change in grip strength	
BDNF and Noggin	Adenoviral-mediated expression (~1.5 × 10^9^ vector genomes each)	R6/2 (HD)	✓	Improved motor phenotype (rotarod and open field activity); increased neurogenesis; prolonged life span	Cho et al. 2007 [88]
CNTF	AAV-mediated expression (2.7 × 10^9^ vector genomes)	R6/1 (HD)	✓	Increased body weight loss rate; aggravated rotarod phenotype; aggravated general appearance and behavior; no change in morphology and distribution of striatal cells; no change in aggregate load	Denovan-Wright et al. 2008 [94]
CNTF	Lentiviral-mediated expression	YAC72 (HD)	✓	Reduced hyperactivity; reduced number of striatal dark cells	Zala et al. 2004 [93]
			×	No change in clasping behavior, rotarod phenotype, and brain weight loss; decreased number of DARPP-32 and neun positive neurons; no change in the number of NADPH-d neurons	
FGF-2	Subcutaneous injection (1.5 μg/week)	R6/2 (HD)	✓	Increased neurogenesis; improved rotarod phenotype; reduced tremor; reduced aggregate formation; decreased body weight loss rate; prolonged life span	Jin et al. 2005 [92]
GDNF	AAV-mediated expression (4 × 10^9^ vector genomes)	N171-82Q (HD)	✓	Improved rotarod and clasping phenotype; increased number and volume of striatal neurons	McBride et al. 2006 [89]
			×	No change in striatal volume and number of total striatal inclusion	
GDNF	Intrastriatal injection of 3 × 10^5^ mNPC cells overexpressing GDNF	N171-82Q (HD)	✓	Improved rotarod phenotype; alleviated aggregate formation; reduced neuronal loss	Ebert et al. 2010 [394]
			×	No change in cortical thickness and in number of dopamine neurons; no long-term change in body weight loss rate	
GDNF	Lentiviral-mediated expression	R6/2 (HD)	×	No change in motor phenotype (rotarod, clasping behavior, open field activity); no change in body weight loss rate; no reduction in brain atrophy, neuronal inclusion formation, and cell proliferation in DG	Popovic et al. 2005 [395]
Neurturin	AAV-mediated expression (4 × 10^9^ vector genomes)	N171-82Q (HD)	✓	Improved motor phenotype (rotarod, clasping behavior, gait pattern); reduced neuronal loss	Ramaswamy et al. 2009 [90]
			×	No change in neuronal morphology and aggregate formation; no change in shortened life span	
NGF	Intrastriatal injection of 6 × 10^5^ MSC cells overexpressing NGF	YAC128 (HD)	✓	Improved rotarod and clasping behavior	Dey et al. 2010 [91]
			×	No change in neuronal loss within the striatum	
VEGF	Overexpression or intracerebroventricular infusion of recombinant Vegf (2.5 μg)	154Q/2Q (SCA1)	✓	Improved rotarod phenotype; increased cerebellar vessel total length and density; increased staining for calbindin	Cvetanovic et al. 2011 [396]

##### BDNF

The brain-derived neurotrophic factor (BDNF) has emerged as the most promising therapeutic neurotrophic factor because it is important in both developing and adult neurons and its expression is deregulated in HD patients and animal models [76, 77]. Normally, BDNF is produced in cortical neurons and is anterogradely transported to the striatum. Mutant huntingtin alters this physiological condition in two ways. First, polyQ mutations affect the normal function of wild-type HTT, which is part of the motor protein complex and promotes vesicular transport along microtubules, subsequently leading to decreased BDNF transportation [78]. Second, mutant huntingtin, through aberrant interactions with transcription factors, can affect the regulation of BDNF promoters, inducing its striatal deficits [79]. Alberch’s group used R6/1 mice with a partial depletion of endogenous BDNF to demonstrate that this protein is involved in the regulation of both the age of onset and the severity of motor and neuronal dysfunctions in vivo [80–82]. Moreover, intrastriatal injection of BDNF in R6/1 animals is sufficient to restore the enkephalin level in striatal projection neurons; this population of neurons is one of the most affected populations in HD [80]. The beneficial effects of increases in BDNF level were observed by other groups and confirmed the results from studies of toxin-induced HD rats that had originally demonstrated the neuroprotective effect of neurotrophins [83–85]. Gharami and colleagues and Xie and colleagues increased BDNF levels by crossing R6/1 and YAC128 animals, respectively, with mice that were overexpressing BDNF under the control of the promoter for the alpha subunit of Ca^2+^/calmodulin-dependent protein kinase II. This strategy resulted in increased levels of BDNF and TrkB signaling activity in the cerebral cortex and striatum, which ameliorated motor dysfunction and rescued brain weight loss [86, 87]. Cho and colleagues used adenoviral vectors to deliver BDNF- and Noggin-encoding constructs to the ependymal cells of R6/2 animals. They observed recruitment of neuronal cells to the adult striatum from subependymal progenitors and the subsequent development of recruited neuronal cells into DARPP-32+ and GABAergic medium spiny neurons. Moreover, treated mice showed improvements in motor performance and lived longer than mock-treated and untreated controls [88].

##### Other Factors

Other neurotrophic factors have also been tested in HD mouse models (Table 2). Kordower’s group induced the overexpression of exogenous glial cell line-derived neurotrophic factor and neurturin in the N171-82Q mouse striatum by using AAV vectors. Injection of both factors resulted in neuroprotection of the injected structures against striatal cell loss, as well as a delay in motor deficit progression [89, 90]. Dey and colleagues transplanted bone marrow mesenchymal stem cells that had been genetically engineered to overexpress nerve growth factor or BDNF into YAC128 mice. Both factors reduced clasping behavior, although BDNF also reduced neuronal loss within the striatum of YAC animals [91]. In R6/2 mice, subcutaneously administered fibroblast growth factor 2 crossed the blood–brain barrier, increased the number of proliferating cells by 150 %, reduced polyglutamine aggregates, improved motor performance, and extended the lifespan [92]. Interestingly, lentiviral and AAV vectors mediating the long-term expression of ciliary neurotrophic factor have not produced beneficial effects in YAC72 and R6/1 mice, respectively. In the second case, the mice developed motor impairments at an earlier age and displayed significant decreases in the levels of striatal transcripts instead [93, 94].

##### Approaches that Involve Inducing the Expression of Endogenous Neurotrophic Factors

Whereas viral-mediated overexpression, transplantation of engineered cells or crossing approaches are useful methods of inducing the expression of neurotrophic factors in laboratory animals; implementing this strategy in humans would require more convenient methods. One possible method would be to enhance the expression of endogenous factors by means of small molecules that are capable of crossing the blood–brain barrier. Antidepressants that belong to the selective serotonin reuptake inhibitor (SSRI) class, such as sertraline, fluoxetine and paroxetine, prolong lifespan, improve motor and neuropathological phenotypes and enhance neurogenesis in the R6 and N171-82Q mouse models of HD. These beneficial effects may be partially mediated by the ability of SSRIs to increase endogenous BDNF levels [95–99]. The positive effect of SSRI treatment may also result from serotonin-induced neuroprotective pathways. Serotonin triggers signaling cascade that lead to neurite outgrowth, synaptogenesis, neurogenesis and cell survival, and BDNF can promote the development and function of serotonergic neurons [100].

BDNF upregulation was reported in several experimental HD treatment approaches, including:

- Modulation of AMPA-type glutamate receptor by ampakine CX929 [101, 102]
- Modulation of signaling pathways, such as JNK and ERK (using CEP-1347), cAMP/CREB (using phosphodiesterase inhibitors rolipram and TP-10), or the Ask1 apoptotic pathway (using an anti-Ask1 antibody) [103–106]
- Strategies aimed at reversing mitochondrial energy impairment [107–109]
- Transglutaminase activity modulation with cystamine and cysteamine treatment [110]
- Dietary restriction [111]
- Anti-excitotoxic drugs memantine and riluzole, which have been used in HD mouse treatment and have also increased BDNF levels in other studies [112, 113]

The finding that BDNF expression and activity are controlled by a complex network that involves many regulatory activities in which polyQ HTT also participates may explain the frequent reports of BDNF upregulation in conjunction with HD therapy [114, 115]. Therefore, using nonselective drugs or targeting signaling pathways that regulate BDNF expression may coincidently result in increased BDNF levels. Although such upregulation is generally positive from a therapeutic point of view, it may be misleading when the molecular mechanisms of the beneficial effects need to be interpreted.

#### Environmental Enrichment

The environmental enrichment strategy is one where the animals are kept in improved environmental conditions relative to standard laboratory housing methods and are provided with objects that promote physical, cognitive, and social development (Fig. 4). The experimental method of enrichment is complex and usually includes more than one of the following: large cages and housing in larger groups, tunnels, nesting materials, toys that are changed frequently and introduced as an element of novelty, and opportunities for physical activity (usually running wheels or treadmills) [116]. The initial reports of the beneficial effect of environmental enrichment in experimental therapy for Huntington disease appeared in 2000 and showed that exposing R6 mice to a stimulating, enriched environment from an early age reduced motor impairment and some of the neuropathological aspects of HD [117, 118]. Subsequently, many groups tested the ability of enriched conditions, dietary enhancement, or voluntary or forced motor training to elicit phenotypic improvements, and these groups found effects that were generally positive but rather moderate [28, 119–129] (Table 3). The mechanisms that account for such a rescue of neuropathological and motor functions are not fully understood. Environmental manipulations result in the upregulation of genes involved in synaptic plasticity and growth, including neurotrophic factors and neurotransmitters, which, as a result, may lead to general improvement in the health of the nervous system due to increased neuroprotection and neurogenesis [130–132]. In R6/1 studies, Spires and colleagues reported increased BDNF levels in the mouse hippocampus and striatum [121], Glass and colleagues showed that an enriched environment reduced the depletion of cannabinoid CB1 receptors [120], and Lazic demonstrated that improved conditions may affect neurogenesis [122]. However, recent reports using microarray profiling have not confirmed the previous observations and have shown no specific changes in enrichment-related gene expression in either transgenic or wild-type mice. In the same work, a nonsignificant trend toward the preservation of downregulated neurotransmitter receptors in the striatum of environmentally enriched mice was observed [126]. Similarly, motor training does not enhance hippocampal neurogenesis in R6/2 mice and does not rescue the deficits of BDNF expression in R6/1 mice [123, 133]. The complex character of the environmental modulation methods, consisting of many unspecified motor and cognitive stimuli whose effects may overlap and act synergistically, may generate numerous variables that influence the experimental outcome. Further studies separating the individual elements of environmental enrichment are required to assess their contributions to the overall effect and determine the relevant mechanisms.

**Table 3:** Environmental enrichment-related therapeutic approaches in mouse models of polyQ diseases

Approach	Model		Therapeutic outcomes	Reference
Environmental enrichment cages (exercise wheels, hiding tubes, and social interaction)	N171-82Q (HD)	✓	Improved rotarod performance; attenuated body weight decline	Schilling et al. 2004 [28]
		×	No change in life span	
Environmental enrichment cages (cardboard, paper, and plastic objects)	R6/1 (HD)	✓	Delayed “turning task” phenotype and clasping behavior; attenuated peristriatal cerebral atrophy	van Dellen et al. 2000 [117]
		×	No change in body weight loss rate and no significant change in striatal volume; no significant difference in the overall density of inclusions	
Environmental enrichment cages (cardboard boxes, open wooden boxes,cylindrical cardboard tunnels, and folded sheets of paper)	R6/1 (HD)	✓	Improved rotarod performance; partially ameliorated body weight loss; increased striatal BDNF level; increased cortical DARPP-32 level	Spires et al. 2004 [121]
		×	No improvement in brain weight loss; no change in striatal DARPP-32 level	
Voluntary physical exercise (running wheels)	R6/1 (HD)	✓	Reduced abnormal rearing behavior; delayed rear paw clasping behavior; rescued deficit in spatial working memory; increased striatalmRNA *BDNF* level	Pang et al. 2006 [123]
		×	No change in rotarod performance; no change in abnormal BDNF levels	
Environmental enrichment cages (plastic and cardboard objects)	R6/1 (HD)	✓	Increased number of BrdU + amd DCX + cells in dentate gyrus; increased length of neuritis; increased DCX + cells migration distance from subgranular zone	Lazic et al. 2006 [122]
		×	No change in the number of BrdU + cells in subventricular zone; no change in rotarod performance	
Environmental enrichment cages (cardboard boxes, plastic conical tubes, cylindrical cardboard tunnels, and folded sheets of paper)	R6/1 (HD)	✓	Improved performance on accelerating rotarod rescued abnormal habituation of locomotor activity and exploratory behavior	van Dellen et al. 2008 [125]
		×	No change in body and brain weight loss; no reduction in shrinkage of the striatum and anterior cingulate cortex; no change in density of protein aggregates	
Voluntary physical exercise (running wheels)	R6/1 (HD)	✓	Delayed horizontal rod phenotype and clasping phenotype; rescued abnormal habituation of locomotor activity and exploratory behavior	van Dellen et al. 2008 [125]
		×	No change in performance on accelerating rotarod; no change in body and brain weight loss; no reduction in shrinkage of the striatum and anterior cingulate cortex; no change in density of protein aggregates	
Environmental enrichment cages (objects varying in shape, texture and size); more frequent animal handling	R6/1 (HD)	✓	Ameliorated deficit in spatial learning on the Barnes maze; increased cortical and hippocampal synaptophysin levels; increased hippocampal PSD-95 level	Nithianantharajah et al. 2008 [124]
Environmental enrichment cages (novel objects)	R6/1 (HD)	✓	Reduced accumulation and size of NII	Benn et al. 2010 [126]
		×	No change in dopamine and adenosine receptor binding levels; no significant environmental enrichment-related changes detectable by microarray; no difference in the level of transgene mRNA expression	
Environmental enrichment cages (cardboard rolls, wire, mesh, shredded paper, wooden, and plastic objects)	R6/1 (HD)	✓	Altered methylation pattern at specific sites within CpG islands	Zajac et al. 2010 [128]
		×	No change in hippocampal *BDNF* mRNA level in R6/1 mice	
Voluntary physical exercise (running wheels)	R6/1 (HD)	✓	Increased hippocampal *BDNF* mRNA levels (females only); altered methylation pattern at specific sites within CpG islands	Zajac et al. 2010 [128]
Enhanced diet + mixed housing of TG mice with WT mice	R6/2 (HD)	✓	Decreased body weight loss rate; increased in the survival of the first 50 % of mice to die	Carter et al. 2000 [118]
Enhanced diet + early weaning and behavioral testing	R6/2 (HD)	✓	Increased in the survival of the first 50 % of mice to die	Carter et al. 2000 [118]
		×	No change in body weight loss rate	
Enhanced diet + involvement in a breeding program	R6/2 (HD)	✓	Increased in the survival of the first 50 % of mice to die	Carter et al. 2000 [118]
		×	Increased body weight loss rate	
Enhanced diet	R6/2 (HD)	✓	Decreased body weight loss rate; prolonged life span; increased hind limb grooming and burrowing	Carter et al. 2000 [118]
		×	No change in open field phenotype	
Minimally enriched living conditions (food pellets on the cage floor + a cardboard tube	R6/2 (HD)	✓	Increased rotarod performance; not significant trend toward increase of the grip strength	Hockly et al. 2002 [119]
		×	No change in body weight loss rate; no change in brain weight loss	
Highly enriched living conditions (larger cages, mixed genotypes, maize fibers, paper strips, cellulose pads, and cotton wool; running wheels and other toys)	R6/2 (HD)	✓	Increased rotarod performance; increased grip strength at endpoint; not significant trend toward increase of the striatal volume; increased peristriatal cerebral volume	Hockly et al. 2002 [119]
		×	No change in body weight loss rate; no change in brain weight loss; no change in striatal and cortical aggregate densities	
Voluntary physical xercise (running wheels)	R6/2 (HD)	×	No change in proliferation of hippocampal cells in R6/2 mice; no change in number of neural precursor cells (DCX+) in the DG of R6/2 mice; no change in the total number of newly generated neurons	Kohl et al. 2007 [133]
Environmental enrichment cages (playground/no handling)	R6/2 (HD)	✓	Increased activity; prolonged life span	Wood et al. 2010 [129]
		×	No change in overall cognitive performance of R6/2 mice in morris water maze (sex-dependent improvement in some tasks); sex-specific mix of beneficial and detrimental effects on body weight loss	
Environmental enrichment cages (playground/ handling)	R6/2 (HD)	✓	Increased activity	Wood et al. 2010 [129]
		×	No change in overall cognitive performance of R6/2 mice in morris water maze (sex-dependent improvement in some tasks); sex-specific mix of beneficial and detrimental effects on body weight loss; shortened life span (males)	
Motor stimulation (enforced physical exercise on the rotarod)	R6/2 (HD)	✓	Increased rotarod performance (females only)	Wood et al. 2011 [127]
		×	Decreased body weight loss rate; no change in survival; no change in cognitive function (Lashley maze performance)	
Cognitive stimulation (training in the OX maze)	R6/2 (HD)	✓	Increased cognitive function in males (Lashley maze performance); prolonged life span (males); sex-specific mix of beneficial and detrimental effects on body weight loss; increased rotarod performance (females)	Wood et al. 2011 [127]
Mixed stimulation (access to a playground)	R6/2 (HD)	✓	Increased rotarod performance	Wood et al. 2011 [127]
		×	No change in body weight loss rate; no change in cognitive function (Lashley maze performance); shortened life span (males)	

### Target: Aberrant Neurotransmission and Excitotoxicity

The hypothesis that a pathogenic mechanism in HD may depend on excitotoxic neuronal damage arose from experiments where exogenous excitotoxins, such as kainic acid and quinolinic acid (QUIN), were applied by direct injection into the healthy rodent striatum, which produced a behavioral phenotype and cell damage pattern that were reminiscent of HD (for review [134]). Quinolinic acid, a selective *N*-methyl-d-aspartate (NMDA) receptor agonist, is useful because its injection selectively affects medium spiny neurons (MSNs), and most of the interneurons remain intact [135]. Excitotoxic neuronal injury in response to the injection of quinolinic acid affects cells that have NMDA receptors (NMDARs) on the cell membrane; these cells are physiologically excitable and may be considered healthy neuronal cells. Moreover, the excitotoxic injury in the QUIN model happens in healthy striata where the connectivity of the corticostriatal pathway is intact (developing young animals where the corticostriatal pathway is not fully established are resistant to QUIN excitotoxicity [136]). This chemical QUIN-evoked model was used to investigate HD in the absence of an identified causative gene and a lack of genetic mouse models, and although these experiments were performed almost 30 years ago, why MSNs are selectively vulnerable in HD is still unknown. Once the R6/2, YAC72, and YAC128 models were generated, they revealed some aspects of the excitotoxic phenomenon, showing electrophysiological alterations in MSNs and cortical pyramidal neurons [137–144]. The electrophysiological changes in HD are biphasic between presymptomatic and symptomatic HD phases and have opposite characteristics between MSNs and pyramidal neurons, and these changes include both excitatory and inhibitory events. Based on these findings, it has been proposed that the connectivity in the corticostriatal pathway is disrupted in HD [145]. A sign of this connectivity loss is the lack of an excitotoxic response in symptomatic R6/2 and YAC128 models following the injection of QUIN [137].

Excitotoxicity is initiated by a glutamate receptor-mediated, excessive influx of Ca^2+^ ions into the neuronal cells, which subsequently may lead to the cascade of destructive events. It is still elusive why neurons that express a polyglutamine protein are more vulnerable to the endogenous excitotoxic insult. One possible explanation is that deregulation of the kynurenine pathway leads to elevated levels of endogenous excitotoxins [146]. Alterations in the metabolism and transport of glutamate or the oversensitivity of glutamate receptors in affected neurons may also contribute to the observed vulnerability [134].

#### NMDA Receptors

Although the central role of NMDA receptors in pathogenesis of HD is well established [147], the receptors were deemed poor therapeutic targets because of their essential physiological role. However, it has been recently discovered that the extrasynaptic pool of NMDARs mediates the deleterious effects of glutamate in neurons, whereas synaptically localized NMDA receptors do not induce Ca^2+^ overload or toxic cellular effects [148–151]. Two studies have demonstrated that selectively blocking extrasynaptic NMDA receptors with low concentrations of memantine, an NMDAR blocker, may represent a novel therapeutic strategy for HD; these studies have also shown that high concentrations of memantine block all NMDA receptors and do not produce beneficial therapeutic effects in transgenic mice [149, 150].

Subcutaneous injection of an NR2B-selective NMDA receptor antagonist does not relieve any disease phenotypes in R6/2 mice [152]. NMDA–NR2B receptors are believed to be located extrasynaptically; however, because the study on R6/2 mice used high doses of NR2B antagonists, the whole pool of NMDA receptors may have been inhibited, which is not beneficial [149, 152]. In addition, the low-affinity NMDA antagonist remacemide has beneficial effects on motor performance in N171-82Q mice but has no effect on survival [28, 153]. Instead, beneficial effects, such as increased survival, have been shown in R6/2 and N171–82Q animals that were treated with remacemide combined with coenzyme Q10 [154].

#### Other Receptors

Other receptors that are present in the membranes of MSNs, such as the adenosine A_2A_ receptor and metabotropic glutamate receptors mGluR5 and mGluR2 may also be possible targets for providing neuronal protection in HD [155–158]. For instance, treatment with CGS21680, an A_2A_ agonist, ameliorates motor and neuropathological phenotypes and reduces hyperglycemia in R6/2 mice [157, 159]. The metabotropic glutamate receptors modulate glutamate-mediated excitotoxicity by controlling membrane enzymes and second messenger systems. The mGluR5 antagonist MPEP and the mGluR2 agonist LY379268 modify disease progression and increase survival in the R6/2 model [160, 161]. Moreover, interfering with glutamatergic neurotransmission by increasing the expression of glutamate transporter protein GLT-1 may be beneficial in HD mouse models [162, 163]. The upregulation of glutamate transporters in astroglia following treatment with PACAP, EGF, or TGF-α presents a potential therapeutic option in neurodegenerative diseases [164, 165]. Other therapeutic options include using reuptake inhibitors or supplying neurotransmitter precursors to interfere with other excitatory and inhibitory neurotransmitters, such as dopamine and GABA, and serotonin [166–169].

#### Kynurenine Pathway

The kynurenine pathway (KP) is a major route of tryptophan catabolism in mammalian cells and contains three important neuroactive metabolites: quinolinic acid, its precursor 3-hydroxykynurenine (3-HK) and kynurenic acid (KYNA) [170]. Because of their properties—the potent excitotoxicity of QUIN, the generation of reactive oxygen species by 3-HK, and the neuroprotective abilities of KYNA—all of these compounds may participate in HD pathogenesis [146, 171, 172]. For detailed mechanisms of neurodegeneration associated with kynurenine pathway see the following references [134, 146].

Therapeutic interventions that modulate the production of KP metabolites have recently been shown to have beneficial effects in a drosophila model of Huntington disease [173] and in mouse models of Alzheimer disease and Huntington disease [174]. In these reports, researchers inhibited kynurenine 3-monooxygenase (KMO, an enzyme that converts kynurenine into 3-HK), which resulted in the increased synthesis of a neuroprotective KYNA metabolite and decreased extracellular glutamate levels. Interestingly, JM6, a KMO inhibitor that was used in the mouse studies, does not trigger these effects directly in central nervous system (CNS) cells because of its inability to cross the blood–brain barrier. Instead, JM6 inhibits KMO in blood cells, which results in an increased level of circulating kynurenine, active transport of kynurenine through the blood–brain barrier, and the subsequent conversion of kynurenine into KYNA in CNS cells. R6/2 mice that were orally administered JM6 did not exhibit cortical or striatal synaptic losses or inflammatory microglial responses, and they lived longer than untreated R6/2 animals. No changes in the abundance or sizes of huntingtin inclusions were recorded [174].

### Target: Mitochondrial Dysfunction

**Fig. 5:**
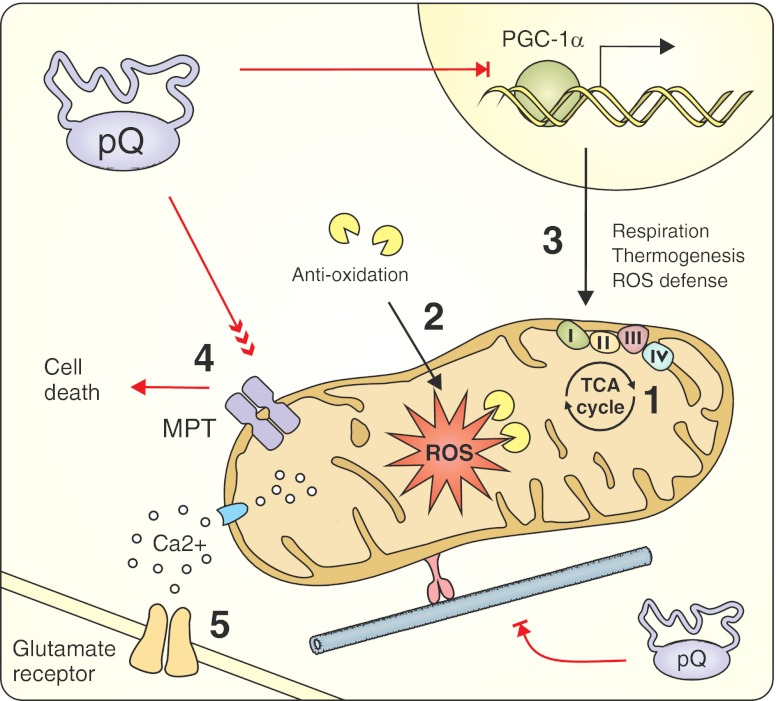
The therapeutic strategies targeting mitochondrial dysfunction induced by expanded polyQ proteins. By interfering with CREB function, huntingtin downregulates the expression of PGC-1α and induces transcriptional deregulation of nuclear-encoded mitochondrial proteins that are involved in respiration, thermogenesis, and ROS defense. Mutant huntingtin has been proposed to interact with the outer mitochondrial membrane to significantly decrease the mitochondrial Ca^2+^ capacity and directly induce MPT pore opening. These alterations can cause increased vulnerability to glutamate receptor-mediated excitotoxic stimuli. Finally, mutant huntingtin causes defects in mitochondrial trafficking through long dendritic and axonal projections. Mitochondria-related therapeutic strategies include compensating for energy deficits (*1*) and oxidative stress (*2*) caused by mitochondrial dysfunction; restoring the altered transcription of mitochondrial factors (*3*); inhibiting the mitochondrial permeability transition (*4*); and administering NMDAR inhibitors to protect against excitotoxicity-mediated cell death (5). PPARγ co-activator-1α (*PGC-1α*), reactive oxygen species (*ROS*), mitochondrial permeability transition (*MPT*)

Numerous observations in postmortem HD brains and animal models of HD support the idea that mitochondrial impairment may contribute to the pathogenesis and neurotoxicity of HD [175–179]. Subsequent studies revealed the existence of several different mechanisms that directly or indirectly link mitochondrial dysfunction with the mutant huntingtin protein (Fig. 5). Among them, the most extensively studied and the most plausible mechanisms are: transcriptional deregulation of nuclear-encoded mitochondrial proteins, Ca^2+^ handling impairment, and trafficking deficits.

Mutant huntingtin induces transcriptional deregulation of nuclear-encoded mitochondrial proteins by binding to several transcription factors, such as p53, CBP, TAFII130 and SP1, and altering their physiological functions. By interfering with CREB function, huntingtin downregulates the expression of PPARγ co-activator-1α (PGC-1α), thus deregulating the expression of numerous proteins that are essential for proper mitochondrial operation, including reactive oxygen species (ROS)-scavenging enzymes, respiratory chain components, and thermogenic factors [180, 181].

When the mitochondrial Ca^2+^-buffering capacity is overloaded, mitochondria lose their membrane potential and open the mitochondrial permeability transition (MPT) pores, which results in the activation of cellular death pathways [182]. Mutant huntingtin may interact with the outer mitochondrial membrane and directly induce MPT pore opening. Furthermore, mutant huntingtin significantly decreases the Ca^2+^ threshold necessary to initiate this cascade [183]. Increased mitochondrial sensitivity to intracellular calcium concentrations may therefore explain the excitotoxicity-mediated death of neuronal cells that contain mutant huntingtin.

Neurons with long dendritic and axonal projections are particularly dependent on proper trafficking of mitochondria to distant energy-consuming sites. One of the normal functions of wild-type huntingtin is interacting with numerous trafficking mediators to regulate intracellular microtubule-mediated transport [184]. It has been demonstrated that mutant huntingtin can negatively influence the trafficking regulation network and cause defects in organelle movement [185]. In addition, large intracellular aggregates of mutant huntingtin can physically block the transport of mitochondria along these projections [186].

#### Energy Deficit and Oxidative Stress

Mitochondria-related therapeutic strategies that have been examined in mouse models of Huntington disease were primarily designed to compensate for energy deficits and oxidative stress caused by mitochondrial dysfunction (Fig. 5). Several studies tested coenzyme Q10 (CoQ10), an essential cofactor of the electron transport chain and potent free radical scavenger, both alone and in multidrug therapies. Schilling and colleagues evaluated the effects of CoQ10 and the excitotoxic protector remacemide on the phenotype of N171-82Q mice. They observed an improvement in rotarod performance and a rescue from body weight loss but not from premature death [28, 153]. The Beal’s and Ferrante’s groups also detected significant rescue of motor impairment and neuropathology in addition to extensions of the lifespans of R6/2 and N171-82Q animals by as much as 20–30 % upon treatment with CoQ10 alone [187] or in conjunction with remacemide [154], minocycline [188], or creatine [189] (Table 4). However, a recent paper by Menalled and colleagues questioned the beneficial effects of CoQ10 (and minocycline; see “Target: Apoptosis” section) by showing a lack of improvement in survival, body weight, rotarod performance, open field performance, and climbing performance following oral administration of 0.2 % CoQ10. Treatment with a higher dose (0.6 %) had additional negative effects that were specific to HD (Table 4) [190]. These contradictory results may have been caused by methodological variations in testing paradigms and by differences in animal husbandry like access to food or housing conditions. Additionally, high molecular weight, insolubility in water, and limited solubility in lipids are reasons for the poor absorption and brain penetration of orally administered CoQ10, so its bioavailability is strongly dependent on the formulation [191]. Various formulation strategies for improving CoQ10 bioavailability are currently under intensive development [192].

**Table 4:** Therapeutic approaches using CoQ10 in mouse models of polyQ diseases

Drug	Route/Dose	Model		Therapeutic outcomes	Reference
CoQ10/remacemide	Food supplemented with 0.2 % of CoQ10 (500 mg/kg/day) and 0.007 % of remacemide (17.5 mg/kg/day)	N171-82Q (HD)	✓	Improved rotarod performance; decreased body weight loss rate	Schilling et al. 2001 [153]
			×	No change in survival; no change in inclusion formation	
CoQ10	Powdered food supplemented with 0.2 % of CoQ10 (500 mg/kg/day)	N171-82Q (HD)	✓	Improved rotarod performance	Schilling et al. 2004 [28]
			×	Shortened life span (powdered food formulations effect)	
CoQ10/remacemide	Food supplemented with 0.2 % of CoQ10 (400 mg/kg/day) and 0.007 % of remacemide (14 mg/kg/day)	N171-82Q (HD)	✓	Attenuated body weight loss; prolonged life span	Ferrante et al. 2002 [154]
CoQ10/remacemide	Food supplemented with 0.2 % of CoQ10 (400 mg/kg/day) and 0.007 % of remacemide (14 mg/kg/day)—separate or combined	R6/2 (HD)	✓	Improved rotarod performance; attenuated body weight loss; prolonged life span; delayed brain weight loss; attenuated gross brain atrophy and ventricular enlargement; attenuated neuronal atrophy; reduced number of striatal aggregates	Ferrante et al. 2002 [154]
CoQ10	Food supplemented 1,000, 5,000, 10,000, or 20,000 mg/kg/day (Chemco)	R6/2 (HD)	✓	Prolonged life span (dose dependent); reduced body weight loss; improved rotarod phenotype; increased forelimb strength; attenuated gross brain size decline and striatal atrophy; reduced aggregate formation	Smith et al. 2006 [187]
CoQ10	Food supplemented 400, 1,000, and 2,000 mg/kg/day (Tishcon)	R6/2 (HD)	✓	Prolonged life span (dose dependent); reduced body weight loss; improved rotarod phenotype	Smith et al. 2006 [187]
CoQ10/minocycline	Food supplemented with 0.2 % of CoQ10 and intraperitoneal injection of minocycline (5 mg/kg/day)—separate or combined	R6/2 (HD)	✓	Prolonged life span; improved rotarod phenotype; reduced body weight loss (CoQ10 specific); attenuated gross brain atrophy and ventricular enlargement; attenuated neuronal atrophy; reduced aggregate formation (CoQ10 specific); attenuated the microglial response (minocycline specific)	Stack et al. 2006 [188]
CoQ10/creatine	Food supplemented with 1 % of CoQ10 and 2 % of creatine—separate or combined	R6/2 (HD)	✓	Improved rotarod performance; prolonged life span	Yang et al. 2009 [189]
CoQ10	Food supplemented with 0.2 % of CoQ10	R6/2 (HD)	×	No change in survival, body weight and rotarod performance; no change in rearing frequency and climbing performance; transient deleterious effects in the open field and grip strength;	Menalled et al. 2010 [190]
CoQ10	Food supplemented with 0.6 % of CoQ10	R6/2 (HD)	×	No change in survival, rotarod performance, grip strength performance, and climbing; transiently decreased body weight, locomotor activity, and rearing in R6/2	Menalled et al. 2010 [190]

Other energy compensators, such as dichloroacetate and lipoic acid (which are pyruvate dehydrogenase complex stimulators), triacetyluridine, creatine, or modified diet regimes, also have beneficial effects on motor and neuropathological phenotypes, prolong the shortened lifespan and rescue body weight loss in R6 and N171-82Q mice [107, 111, 118, 193–198].

Damaged mitochondria lose their free radical scavenging properties, which leads to elevation of free radical concentration [199]. To mitigate this aspect of HD pathogenesis, several antioxidant approaches have been extensively investigated. In addition to having anti-oxidative properties, the chemical compounds NDGA and TUDCA also mitigate mitochondrial insufficiency and toxicity and improve the phenotype of R6/2 mice [200, 201]. Other potent free radical scavengers, such as BN82451, ascorbate and l-carnitine, or triterpenoids and fumaric acid, which indirectly stimulate the Nrf2 antioxidative signaling pathways, reduce ROS and have a high therapeutic potential for HD treatment [202–206].

#### Transcriptional Deregulation of Mitochondrial Proteins

Approaches that restore the altered transcription of mitochondrial factors target the activity of PGC-1α, a key transcriptional co-activator involved in energy homeostasis, glucose metabolism, and mitochondrial biogenesis [180]. Chaturvedi and colleagues mimicked the effects of endurance exercise training by treating NLS-N171-82Q mice with GPA (beta-guanidinopropionic acid), which reduces phosphocreatine and ATP levels. They found that muscles in HD mice cannot overcome an energetic stress by inducing the PGC-1α pathway, as it happens in muscles of wild-type mice. This deficiency is caused by alterations located upstream of PGC-1α that impair the activation of the PGC-1α-inducing AMPK pathway, the sensor for energy regulation. Expression of exogenous PGC-1α directly in the muscles of NLS-N171-82Q mice increases the oxidative capacities of the muscles and reverses the blunted response to GPA treatment [207].

PGC-1α activity is also regulated through its deacetylation by NAD-dependent deacetylase sirtuin-1 (Sirt1). Sirt1 activity can be enhanced by resveratrol, a natural polyphenolic compound. Ho and colleagues found that orally administered resveratrol increased both PGC-1α activity and the expression of its direct downstream targets, NRF-1 and UPC-1 (both of which regulate mitochondrial function) in N171-82Q transgenic mice. However, this improvement was only observed in brown adipose tissue. As a result of insufficient striatal penetration, resveratrol could not induce similar effects in neurons; thus, the treatment resulted in a lack of improvement in motor performance, survival, and striatal atrophy [208]. A similar strategy, leading to an increase in PGC-1α activity, was applied by Hathorn and colleagues. They used nicotinamide, an Sirt1 inhibitor, and postulated that it can also act as an Sirt1 activator. In contrast to Ho’s report, nicotinamide improved HD-associated motor deficits [108]. However, because nicotinamide is not a specific drug (it can also increase BDNF expression), PGC-1α-independent mechanisms may also contribute to the observed improvement. Recently, two studies showed that Sirt1 exerts neuroprotection in HD mouse brains by activating pro-survival transcription factors/coactivators, including Foxo3a and TORC1. Mutant huntingtin interacts with Sirt1 and inhibits its deacetylase activity, which results in hyperacetylation of Sirt1 substrates and repressed transcription of pro-survival genes. Notably, Sirt1 overexpression restores the aberrant acetylation status of Sirt1 substrates, promotes the BDNF and DARPP32 expression, and improves HD phenotype in N171-82Q and BACHD mice [209, 210].

Finally, Chiang and colleagues showed that treatment of R6/2 mice with thiazolidinedione to activate pathways mediated by PPARγ (a nuclear receptor that acts upstream of the PGC-1α gene), can rescue the progressive weight loss, deterioration of motor skills, formation of mutant HTT aggregates and reduced lifespan phenotypes. Similar to nicotinamide treatment, thiazolidinediones also induce the expression of two neuroprotective proteins, BDNF and Bcl-2 [109].

#### Calcium-buffering Capacity

The importance of mitochondrial permeability in the transition to cell death has been tested twice in R6/2 mice. Administration of nortriptyline, a strong inhibitor of MPT, delays disease onset but also accelerates disease progression once the phenotype appears [211]. In addition, Perry et al. obtained discouraging results when they crossbred R6/2 mice with cyclophilin D (CypD)-deficient animals. CypD is a structural component of the MPT pore, and knockout of the gene encoding CypD increases mitochondrial Ca^2+^ buffering, thereby protecting cells from calcium overload. However, increasing the mitochondrial Ca^2+^ capacity fails to ameliorate the HD-related behavioral and neuropathological phenotypes [212].

### Target: Apoptosis

**Fig. 6:**
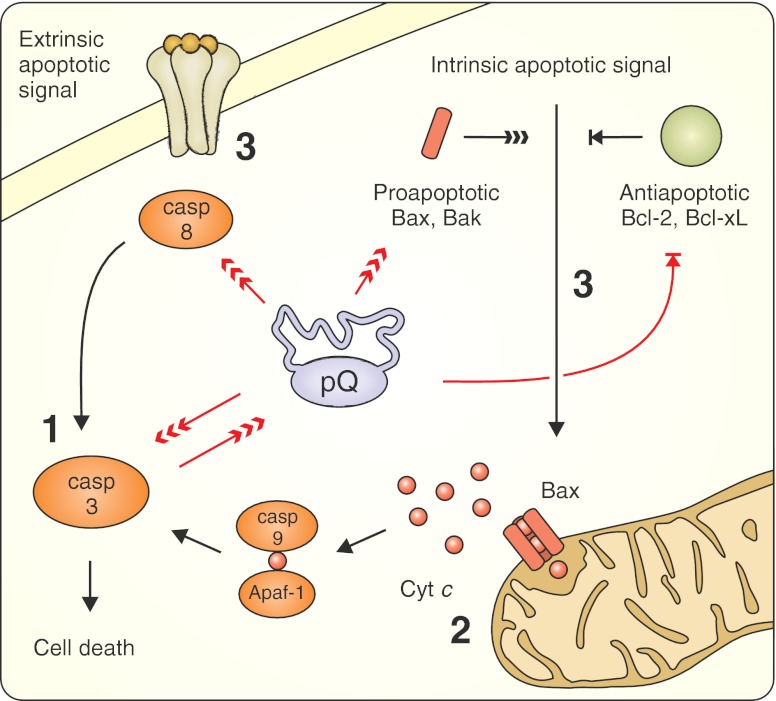
Anti-apoptotic therapeutic strategies target aberrant interactions between polyglutamine proteins and the components of apoptotic pathways. PolyQ proteins cause the upregulation and/or activation of several caspases, upregulate pro-apoptotic proteins and downregulate anti-apoptotic factors, which may subsequently lead to the release of apoptogenic proteins from mitochondria. Several polyQ proteins undergo proteolytic cleavage by caspases, which results in the production of toxic truncated protein fragments. Therapeutic approaches tested in mouse models include the inhibition of caspase functions (*1*), the inhibition of mitochondrial release of cytochrome *c* and subsequent intrinsic apoptotic pathway activation (*2*), and the modulation of the initiation of apoptotic signals (*3*). Cytochrome c (*Cyt c*), apoptotic protease activating factor 1 (*Apaf-1*)

Aberrant interactions between polyQ proteins and the components of apoptotic pathways have been reported in patients’ brains and in cellular and animal models (Fig. 6). The presence of mutant huntingtin results in mitochondrial cytochrome c release, followed by the activation of caspases 9 and 3 and the upregulation and/or activation of caspases 1, 2, 3, 6, 7, and 8 in the brains of humans with HD and in mouse models of HD [213–218]. Expanded ataxin-3 and ataxin-7 upregulate Bax and PUMA (which are pro-apoptotic proteins) and downregulate Bcl-xL (which is an anti-apoptotic protein), which may subsequently lead to the release of the apoptogenic proteins from mitochondria [219–221]. Moreover, the expression of androgen receptors in cultured neurons induces the Bax-dependent apoptotic cascade initiated by the JNK signaling pathway in response to polyQ-mediated stress [222]. These observations suggest that polyQ proteins may interfere with both the intrinsic (mitochondria-mediated) and extrinsic (receptor-mediated) apoptotic pathways. Additionally, several polyQ proteins that undergo proteolytic cleavage by cellular proteases, such as huntingtin, ataxin-3 and -7, AR and atrophin-1, are also substrates for caspases [223–227]. Truncated fragments of these proteins may play crucial roles in the pathogenesis of each disease because they are more toxic than their full-length forms. For example, huntingtin containing a mutation at the caspase-6 cleavage site was unable to induce neurodegeneration in HD YAC128 transgenic mice [228]. The mice expressing the N-terminal 586 aa caspase fragment of HTT show cytoplasmic inclusions and neurological phenotype milder than R6/2. This indicates that further cleavage is needed to worsen the phenotype and evoke more intensive nuclear accumulation [229, 230].

Although molecular markers of apoptosis are easily detectable even before gross neuronal loss, typical cellular apoptotic features and the presence of neurons undergoing apoptosis have rarely been reported in mouse models. It is widely accepted that expanded polyglutamine proteins may also induce other forms of cellular death [231]. Differences in spatiotemporal expression patterns and polyQ protein contexts between humans and mouse models may influence the proportion of individual death mechanisms that are activated and, as a result, apoptosis may be masked in polyQ mice, even with the activation of apoptotic mediators.

#### Minocycline Treatment

The efficacy of minocycline in Huntington disease treatment has been extensively debated over the last decade. Initial encouraging results were obtained by Ona and colleagues who showed that blocking caspase function could delay both disease onset and mortality in R6/2 mice [232]. The same group found that minocycline inhibits caspase-1, caspase-3, and iNOS activities in HD animals and observed a reduction in both disease progression and mortality of R6/2 animals [214]. The beneficial effect of minocycline was thought to be caused by the attenuation of HD-mediated induction of the caspase cascade and decreased production of toxic HTT fragments [214, 233]. However, Bates’s laboratory was unable to replicate those findings despite using the same drug and mouse model but with different dosing and different administration routes [27]. Subsequent studies performed on R6/2, N171-82Q, and 3-nitroproprionic acid HD models demonstrated that minocycline treatment had either favorable efficacy [188] or no efficacy [234, 235] (Table 5). Recently, Menalled and colleagues attempted to reevaluate the preclinical effects of minocycline (and coenzyme Q10) using the R6/2 model. A low dose of minocycline (Table 5) induced some transient beneficial effect, although it was not comparable to the results published by Chen and colleagues and Stack and colleagues. Higher doses (Table 5) resulted in HD-specific toxicity (including reduced survival rates and body weights) and lack of amelioration of the disease phenotype [190], which confirmed previous observations [27]. The reason for these discrepancies is unclear and may be explained by differences in husbandry conditions, drug preparation, or methodological variations in testing paradigms [190, 236].

**Table 5:** Therapeutic approaches using minocycline in mouse models of polyQ diseases

Drug	Route/Dose	Model		Therapeutic outcomes	Reference
Minocycline	Intraperitoneal, 5 mg/kg/day	R6/2 (HD)	✓	Prolonged life span; improved rotarod performance	Chen et al. 2000 [214]
				No change in body weight loss rate and blood glucose level; no change in aggregate formation and receptor-binding	
Minocycline	1 and 5 mg/mL in drinking water, (~150 and 750 mg/kg/day)	R6/2 (HD)	✓	Reduced elevated glucose levels	Smith et al. 2003 [27]
				No change in body weight loss rate, rotarod performance, and grip strength; no change in aggregate formation; higher dose (10 mg/mL) induced severe initial weight loss	
Minocycline	Intraperitoneal, 5 mg/kg/day	R6/2 (HD)	✓	Prolonged life span; improved rotarod performance; attenuated gross brain atrophy and ventricular hypertrophy; attenuated striatal neuronal atrophy and microglial response; therapeutic effect increased with the combined minocycline/CoQ10 treatment	Stack et al. 2006 [188]
			×	No change in body weight loss rate and aggregate formation	
Minocycline	Intraperitoneal, 10 mg/kg/day	N171-82Q (HD)	×	No change in survival and body weight loss rate; no change in rotarod and open field performance; no change in striatal atrophy, ventricle enlargement, and cortical thickness	Mievis et al. 2007 [235]
Minocycline	Intraperitoneal, 5 mg/kg/day	R6/2 (HD)	✓	Transiently increased body weight, locomotor activity, and rearing (males)	Menalled et al. 2010 [190]
			×	No change in survival, grip strength, rotarod performance, and climbing phenotype;	
Minocycline	Food supplemented with 0.1 % and 0.375 % of minocycline (~200 and 750 mg/kg/day)	R6/2 (HD)	✓	Transiently increased body weight and rearing (0.1 %); minor and transient beneficial effect on rotarod performance (0.375 %)	Menalled et al. 2010 [190]
			×	Decreased survival (both doses); reduced body weight and rearing (0.375 %); decreased open field activity (0.375 %); no change in grip strength	

At the same time, four minocycline clinical trials were being conducted. A small study of 14 patients showed stabilization of general motor and neuropsychological function after 2 years of treatment [237]. Another short-term pilot study, designed to examine the safety of orally administered minocycline at the dosage of 200 mg/day, revealed that although there was a lack of medication-related side effects, there was also no significant motor improvement [238]. Similar results were obtained by the Huntington Study Group, although minocycline at 100 and 200 mg/day induced a drop in platelet count and increase in blood urea nitrogen that were not clinically relevant [239]. Observed toxicity is consistent with other studies in humans where low doses of minocycline show no or minor toxicity, and high-dose treatment results in negative effects [240]. In ALS patients, dosages as high as 400 mg of minocycline/day increased mortality, gastrointestinal, and neurological adverse events [241]. Recent results from a futility study (which is used to determine whether phase III efficacy studies should be pursued) suggest that minocycline is ineffective in the treatment of HD [242].

#### Other Treatment Targeting Apoptotic Pathway

Methazolamide has been isolated in an in vitro screen from the library of 1,040 compounds for inhibitors of mitochondrial cytochrome *c* release (and caspase activation). Methazolamide treatment results in the alleviation of motor and neuropathological phenotypes of R6/2 mice [243]. Expression of the P2X7 receptor, an ATP-gated cation channel that may mediate apoptosis in response to elevated Ca^2+^ levels, is increased as a consequence of polyQ-mediated transcriptional deregulation. Thus, inhibition of the P2X7 receptor by Brilliant Blue-G prevents neuronal apoptosis, reduces body weight loss, and improves motor deficits in R6/2 animals [244].

### Target: Transcriptional Deregulation

**Fig. 7:**
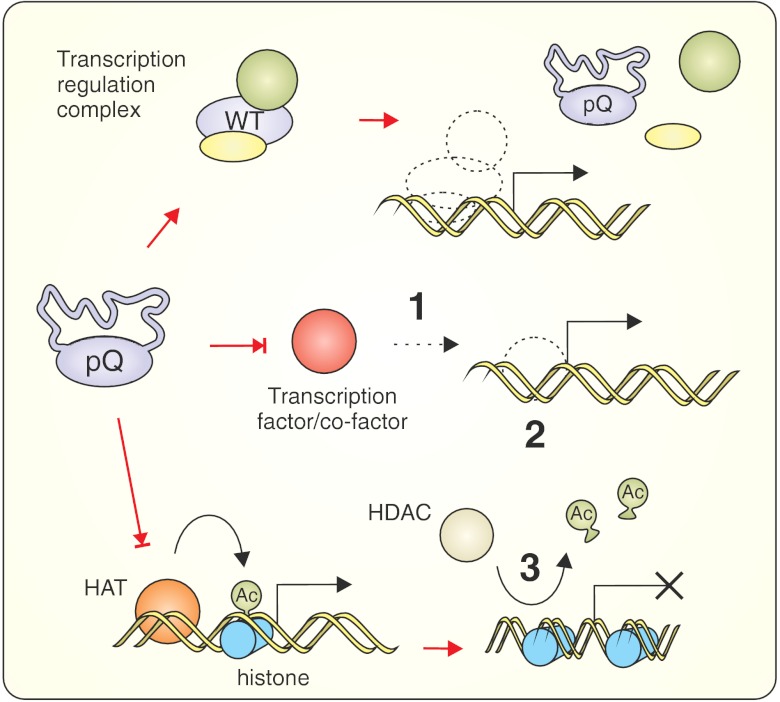
Transcriptional deregulation in therapeutic strategies of polyQ diseases. PolyQ tract may influence the binding of polyQ proteins with other protein partners or DNA response elements. Expanded polyQ stretches may interact with or sequester transcription factors leading to the up- or downregulation of many genes. In particular, mutant polyQ proteins abnormally interact with HATs and/or HDACs, which results in the alteration of histone modification patterns and leads to transcriptional activation or inhibition at specific genomic loci. Therapeutic strategies include activation of transcription factors whose activities are reduced by polyQ proteins (*1*), modulation of transcription factor activity using DNA-binding anthracycline antibiotics (*2*), and restoration of altered transcription patterns through the modulation of nucleosome dynamics using HDAC inhibitors (3). PolyQ protein with normal polyglutamine stretch (*WT*), histone acetyltransferase (*HAT*), histone deacetylase (*HDAC*), acetyl group (*Ac*)

The expression of expanded forms of polyQ proteins leads to transcriptional changes that can be detected in animal models and polyglutamine disease patients [245–249]. Transcriptional deregulation is a common phenomenon that occurs in polyglutamine and other neurodegenerative disorders. Deregulation affects genes that are responsible for neuroprotection and neuronal plasticity; genes that are involved in signaling pathways (including those leading to cellular death); genes that regulate the function of intracellular systems, such as in the mitochondria and in clearance pathways; and genes that are essential for neuronal communication [249]. Therefore, transcriptional aberrations may participate in or even induce other pathological mechanisms, and therapeutic strategies aimed at restoring an altered gene expression pattern may have great potential because they can produce beneficial effects through multiple mechanisms (Fig. 7).

#### Transcription Factors

The polyQ proteins, e.g., the androgen receptor and TATA-binding protein are well-known DNA-binding transcription factors. Moreover, ataxin-7 is a subunit of a STAGA transcriptional coactivator complex [250], and ataxin-1 interacts with and modulates the function of transcriptional coregulators [251–253]. Ataxin-3 is thought to repress transcription via histone-dependent chromatin remodeling [254, 255], and huntingtin modulates the expression of NRSE-controlled genes [79]. The polyglutamine mutations expressed in these proteins change their physiological properties by diminishing or enhancing their abilities to bind other protein partners or by changing their binding to DNA response elements, which ultimately results in the up- or downregulation of many genes. Expanded polyQ stretches may also endow mutant proteins with new abilities to interact with or to sequester transcription factors and cofactors that do not interact with proteins containing normal polyQ tracts (Fig. 7). This mechanism was observed in several studies that reported that polyQ stretches located in various proteins were able to modulate the activities of the transcription factors TAFII130, PQBP-1, p53, and Sp1 [221, 256–259]. The different polyQ proteins often share similar interactions with same transcription factors [249].

The strategy of restoring the activity of transcription factors that were deregulated and/or sequestered by mutant polyQ proteins was applied to a rat HD model and to striatal cell lines established from HdhQ111 knock-in mice. The overexpression of CA150 transcriptional regulator fully rescues the 109Q/109Q striatal cell death in culture and delays striatum shrinkage and the degeneration of striatal cells in the lentiviral rat model of HD [260]. Similarly, an abnormal interaction between mutant huntingtin and Bcl11b, a zinc finger DNA-binding protein, is thought to contribute to the striatal transcriptional deficits that are observed in HD, and overexpression of Bcl11b in STHdh^Q111^ cells partially reverses the expression changes of Bcl11b target genes [261].

#### Chromatin Remodeling

Mutant polyQ proteins often interact abnormally with histone acetyltransferases (HATs) and/or histone deacetylases (HDACs), altering physiological histone modification patterns thereby changing gene expression in the cell (for review [262]). The transcriptional coactivator CREB-binding protein (CBP) contains a HAT domain and interacts with several polyQ mutant causative proteins [263–266]. The inhibition of the acetyltransferase activity of CBP (as well as other HATs, such as p300, PCAF, or TIP60) may lead to hypo-acetylation of histones at several promoters and, consequently, to transcriptional inhibition at specific genomic loci [267].

In healthy cells, cAMP/CREB signaling often leads to the activation of “pro-survival” gene promoters. The polyQ disease mutant proteins may also recruit CREB, preventing the activation of genes. A possible therapy should involve the increase of the intracellular level of cAMP to induce the activation of more CREB molecules. Indeed, the administration of phosphodiesterase (PDE) inhibitors increases cAMP levels by inhibiting its degradation. In R6/2 mice, this kind of treatment using either the PDE type IV inhibitor rolipram [104, 268] or the PDE10E inhibitor TP-10 [105], improves the disease phenotypes.

##### HDAC Inhibitors

The inhibition of HDAC activity appears to be the transcription–restoration strategy that has been most extensively tested in mouse models of polyQ diseases. Although HDAC inhibitors lack specificity because they can also disturb the expression of other, unrelated, genes, some of these inhibitors display promising therapeutic properties. Aliphatic acids, such as butyric, phenylbutyric and valproic acids, administered as sodium salt solutions, have been analyzed in SCA3 Q79, SBMA AR-97Q, DRPLA Atro 118Q and N171-82Q, and R6/2 HD animals [269–274]. These compounds are generally beneficial, restore the hypo-acetylation phenotype, and improve motor performance (Table 6). Despite significant improvements in numerous neuropathological phenotypes, sodium butyrate cannot reduce the amount of polyQ aggregates.

**Table 6:** Therapeutic approaches using HDAC inhibitors in mouse models of polyQ diseases

Drug	Route/dose	Model		Therapeutic outcomes	Reference
HDACi 4b	1 g/L in drinking water, (~150 mg/kg/day)	R6/2^300Q^ (HD)	✓	Improved motor phenotype (rotarod performance, clasping phenotype and general locomotion); reduced hunchback posture; attenuated gross brain size decline and striatal atrophy; attenuated body weight decline	Thomas et al. 2008 [276]
			×	No change in aggregate formation	
Phenylbutyrate	Intraperitoneal (100 mg/kg/day)	N171-82Q (HD)	✓	Prolonged life span; attenuated gross brain atrophy, ventricular enlargement, and striatal neuron atrophy;	Gardian et al. 2005 [272]
			×	No change in rotarod performance and aggregate formation	
SAHA	0.67 g/L in drinking water, (~100 mg/kg/day)	R6/2 (HD)	✓	Improved rotarod performance; attenuated neuronal atrophy	Hockly et al. 2003 [275]
			×	No change in grip strength, gross brain atrophy, and aggregate formation; increased body weight loss rate	
SAHA	0.67 mg/mL in drinking water (~100 mg/kg/day)	R6/2 (HD)	✓	Decreased HDAC 2 and 4 protein levels; decreased HDAC 7 and 11 mRNA levels; restored cortical BDNF mRNA level; reduced cortical aggregate load	Mielcarek et al. 2011 [397]
Sodium butyrate	4 and 8 g/L in drinking water (~800–900 mg/kg/day)	AR-97Q (SBMA)	✓	Improved motor phenotype (rotarod, cage activity, gait pattern); ameliorated muscle atrophy and body posture; decreased body weight loss rate; prolonged life span; improved motor neurons and muscle cells morphology	Minamiyama et al. 2004 [270]
			×	No change in aggregate formation and nuclear localization of mutant AR; higher doses (16 and 40 g/L) accelerated the disease onset	
Sodium butyrate	Intraperitoneal (400 or 800 mg/kg/day)	Ataxin-3-Q79 (SCA3)	✓	Improved rotarod phenotype and gait pattern; reversed reduction of locomotor activity; improved Purkinje cell morphology; decreased body weight loss rate; prolonged life span; reduced pelvic elevation and abnormal hunchback posture	Chou et al. 2011 [269]
Sodium butyrate	Intraperitoneal (0.5 and 1.5 mg/kg/day)	Atro 118Q (DRPLA)	✓	Improved motor phenotype (rotarod and grip strength); prolonged life span	Ying et al. 2006 [271]
			×	No change in aggregate formation and nuclear localization of mutant atrophin; no change in somal size of neurons in dentate cerebellar nucleus	
Sodium butyrate	Intraperitoneal (200, 400, 600, 1,200 mg/kg/day)	R6/2 (HD)	✓	Prolonged life span; improved rotarod performance; increased brain weight; attenuated gross brain atrophy; reduced striatal neuron atrophy	Ferrante et al. 2003 [274]
			×	No treatment related reduction of body weight loss; no significant reduction in huntingtin-positive striatal aggregates	
Sodium valproate	Intraperitoneal (100 mg/kg/day)	N171-82Q (HD)	✓	Prolonged life span; improved open field activity	Zádori et al. 2009 [273]
			×	No changes in the striatal dopamine, DOPAC, or HVA levels	

Another HDAC inhibitor, suberoylanilide hydroxamic acid (SAHA), administered orally in drinking water as cyclodextrin complex, strongly improves motor performance on the rotarod apparatus and decreases neuronal atrophy in R6/2 mice. Like sodium butyrate, SAHA rescues the global hypo-acetylation of histones and has no effect on polyQ aggregates [275]. Finally, Thomas and colleagues treated R6/2 mice (300Q) with a benzamide-type HDAC inhibitor, HDACi 4b. In an in vitro test, this compound had lower toxicity than previously tested HDAC inhibitors, and it prevented motor deficits and neurodegenerative processes in vivo even when treatment was begun after the onset of motor deficits. Microarray analysis revealed that HDACi 4b treatment partially restored the expression changes that had been detected in R6/2 300Q brains [276].

##### Other Chromatin Remodeling Approaches

The anthracycline antibiotics mithramycin and chromomycin directly bind to DNA sequences with guanosine–cytosine base specificity and may interfere with binding of transcription factors to DNA, which activates the transcription of the gene encoding ESET (a methyltransferase) [277]. The mithramycin-mediated Sp1 and Sp3 displacement downregulates ESET expression and reduces the hypertrimethylation of histone H3 at lysine 9. In contrast to histone acetylation, histone methylation is associated with decreased transcriptional activity. The beneficial effects of these clinically approved antibiotics were observed in both R6/2 and N171-82Q HD mice. Mithramycin and chromomycin shift the balance from methylation to acetylation of histone H3 (K9), rescue the downregulation of the subset of affected genes, and improve locomotor and neuropathological phenotypes [277–279].

Another broad transcription regulator, lithium, affects a wide range of cellular functions, for example, it increases the levels of anti-apoptotic factors and affects the PKC and Wnt (through GSK3 inhibition) signaling pathways [280–282]. Zoghbi’s group has shown that lithium carbonate mitigates some motor, neuropathological, and cognitive dysfunctions in 154Q knock-in mouse model of SCA1 [283]. In R6/2 HD mice, lithium treatment is associated with improvements in motor function; however, those effects have not been linked to transcriptional rescue [284].

### Other Therapeutic Strategies

Mouse models of polyglutamine diseases have also been used to test therapeutic strategies that do not directly correspond to the categories described above. These other therapeutic strategies include targeting the diabetes that is present in HD mouse models, modulating transglutaminase activity, interfering with testosterone levels, and activity in SBMA models and regulating sleep/wake cycles. The DNA vaccination against the mutant protein was used in R6/2 HD model, and the overexpression of ataxin-11 (a paralog of ataxin-1) was used to enhance aggregate formation in the SCA1 154/2 model [285, 286]. Using Cox inhibitors to target inflammatory pathways has no beneficial and some deleterious effects on the pathogenesis of HD in R6/2 and N171-82Q mice [28, 287].

#### Anti-diabetic Treatment

Diabetes is present in the pathology of HD patients, and elevated levels of blood and urine glucose develop in some but not all animals in the R6/2, R6/1, Hdh(CAG)150, and N171-82Q models [111, 288–290]. Hypoglycemic agents, such as glibenclamide and rosiglitazone, are not effective for treating diabetes in R6/2 mice [291]. The administration of insulin decreases blood glucose levels in mice, but it can be deleterious to them and may impair their survival, which indicates that these animals have dramatic insulin resistance. In humans and in most models of type 2 diabetes, exercise is considered beneficial for health and lifespan [292]. However, intensive physical exercise, such as excessive behavioral testing, may evoke an earlier onset of diabetes and decrease survival in R6/2 mice [291]. Insulin resistance is only partially ameliorated in male R6/2 mice by metformin and is not ameliorated in female R6/2 mice [293]. Exendin-4, the long-acting glucagon-like peptide-1 receptor agonist, ameliorates high blood glucose and improves motor phenotype in N171-82Q animals [294]. Exendin-4 can cross the blood–brain barrier and bind to the receptors in neurons, which promotes cell survival; exendin‐4 also promotes pancreatic beta-cell growth and the production of insulin.

#### Cystamine Treatment

The transglutaminase inhibitor cystamine has beneficial effects in various HD mouse models such as R6/2 [295–297], YAC128 [298], and the R6/1 and Q111 knock-in models [110]. Moreover, Mastroberardino and co-workers demonstrated that the genetic transglutaminase knockout has rescuing effects when combined with the R6/1 HD model [299]. Despite this body of consistent evidence for a significant role of transglutaminase in the pathogenesis of HD, the mechanism of its pathogenic contribution remains unclear. Some evidence of the mechanism was provided by examining the expression of histone H3 (K9) methyltransferase and observing reductions in the hypertrimethylation of H3 in R6/2 mice in the presence of mithramycin and cystamine. This reduction of methylation by cystamine suggested that transglutaminase 2 could be involved in HD pathogenesis by inducing transcriptional deregulation and chromatin remodeling [277]. Moreover, mithramycin, and cystamine act in an additive way, suggesting that multiple and additive mechanisms induce chromatin remodeling [277]. More direct evidence was provided recently, showing that the polyamination of the N-terminal tail of histone H3 by transglutaminase 2 leads to transcriptional repression of genes that are important for energy homeostasis and that are defective in HD. Moreover, the authors demonstrated that TG2 inhibition protects neurons from NMDA toxicity in YAC128 animals [300].

#### Regulation of the Sleep/Wake Cycle

The deregulation of the sleep–wake cycle is deleterious to cognitive function in healthy individuals and can contribute to disease progression in Huntington [301] and SCA disease patients and transgenic animals [302]. Selective loss of orexin neurons in the hippocampus of the R6/2 and SCA3 models leads to sleep disturbances and narcolepsy [303]. Sleep–wake cycles can be normalized in transgenic R6/2 animals by administering alprazolam and modafinil, which are sleep- and wake-promoting agents, respectively [304]. Interestingly, the cognitive function of HD mice improves not only after promoting regular sleep but also after promoting regular waking, with the most beneficial results reached when both drugs are used regularly [304, 305].

#### Testosterone-Related Approaches

Ataxin-3, huntingtin, androgen receptor, and other polyQ proteins are expressed at high levels in the testes [60, 306]. In patients suffering from polyQ diseases, the testes are often atrophic, and the males of transgenic mouse models are sometimes infertile. SBMA is a special disease condition because the causative gene is the androgen receptor, and affected individuals are males or females who have been exposed to testosterone. The effectiveness of testosterone deprivation in SBMA therapy in mice has been confirmed by castration; administration of leuprorelin, a luteinizing hormone receptor antagonist; or by injection of a testosterone receptor antagonist [307–309]. All of these therapies ameliorate the disease phenotype or even reverse it.

In HD, the problem is different from the problem in SBMA because the HD patients suffer from testicular atrophy thus their testosterone levels are decreased by neuroendocrine changes in hypothalamus [310]. To date, no therapeutic interventions supplementing testosterone or enhancing testosterone production in mouse models of HD have been investigated.

## PolyQ Mouse Models in Experimental Therapies

**Fig. 8:**
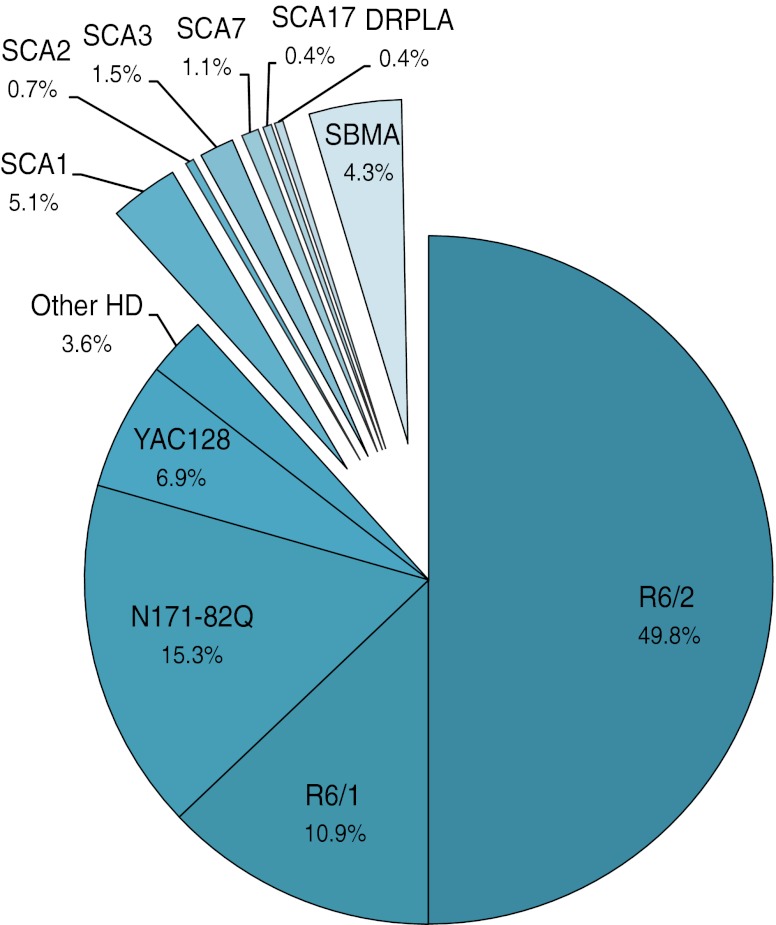
The diagram demonstrates the use of various mouse models in polyglutamine disease therapeutic approaches. The vast majority of these approaches were performed on four Huntington disease models: YAC128, N171‐82Q, R6/1, and R6/2. Overall, Huntington disease animals were used in studies of nearly 90 % of therapeutic approaches (243 of 280). The remaining studies utilized mouse models of SBMA (11 approaches were tested in the AR97Q model and one was tested in the 112Q model), SCA1 (six approaches in the B05 and eight in the 154Q/2Q models), SCA3 (four approaches were tested in four different models—polyQ69, MJD84.2, 70.61CAG, and Q79), and SCA7 (two approaches were tested in the 90QR7E model). In addition, the SCA2 58Q, DRPLA 118Q, and Sca17 L7-hTBP models were also used for experimental therapy

PolyQ experimental therapies are almost exclusively tested in HD mouse models, which were used in tests of approximately 90 % of all approaches (Fig. 8). In addition, more than 80 % of all experimental polyQ therapies were tested in the R6/2, R6/1 [311], and N171-82Q [312] HD models. The remaining studies involve mouse models of SCA1, 2, 3, 7, and 17 (6.4 %); SBMA (4.8 %); and DRPLA (0.4 %). In our previous part I data table (part I of this review) which characterized the polyQ mouse models, we listed over 100 different transgenic animals and their variants. For the present therapeutic data table (Supplementary Material), we found that among nearly 250 experimental therapies, only 21 models were used (Fig. 2).

### HD Fragment Models

The R6/2, R6/1, and N171-82Q are therapeutic models of choice and not always match the specific mechanisms of HD in patients. First, the rapid onset of disease phenotypes observed in these models is rare in patients and is only observed in juvenile forms of HD, the disease symptoms of which differ from adult forms of HD. Second, the mutant HTT proteolysis cleavage step that initiates the pathogenesis of HD may not be required in the R6/2, R6/1, or N171-82Q models because these animals express a transgene protein that is an extremely shortened form of the native protein and that mimics the toxic cleavage fragment. N-terminal cleavage fragments were recently detected in homozygous Hdh(CAG)150 knock-in animals [313]. The putative cleavage of mutant HTT may be a limiting step and may delay pathogenesis in space (not all cell types can cleave HTT) and time (the kinetics of this process may be different in different cell types). These limitations do not exist in the R6/2, R6/1, or N171-82Q mice but may be present in patients. In addition, the behavioral phenotypes that are induced in these animals may result from dysfunction in several brain regions and types of neurons; they may also be more pronounced and less specific than in YAC128 animals. Although the R6/2, R6/1, and N171-82Q models do not imitate HD as it occurs in patients, they exhibit a low degree of variability in tested phenotypes between individual animals. This low variability influences the low standard deviation in experiments and results in a need for fewer animals to record the therapeutic outcome.

### HD YAC Models

The YAC animal models (YAC128, YAC72), which have been used in 22 therapeutic approaches, contain full-length huntingtin with an expanded tract of CAG repeats [314–316]. The mild disease that appears in YAC animals result in slower experimental turnover, and the phenotypes have much greater variability than the phenotypes in R6/2. According to a power analysis performed in Hayden’s laboratory, eight to 35 animals may be required to detect a 33 % change in a tested phenotype, and four to 15 animals may be required to detect a 50 % change in the tested phenotype following the application of a treatment [315]. Rotarod performance is highly variable in YAC128 animals, and 99 and 43 animals would be needed to detect 33 % and 50 % changes, respectively, when testing animals at 6 months of age [315]. These numbers contrast to R6/2 mice, where the experimental cohort can be as small as ten animals for the detection of 10 % changes in some tested phenotypes [317]. Although YAC transgenic animals are not particularly convenient for testing therapeutic approaches, they recapitulate the adult pathogenesis of HD. YAC128 mice show biphasic HD disease, with initial hyperactivity and subsequent hypoactivity that is followed by brain atrophy and neuronal cell loss. Other HD animals that are used in therapeutic approaches include knock-ins containing 140 and 111 CAG repeats [318, 319], the BACHD model [320] and the EGFP HD190QG model [321].

### Other PolyQ Models

Other polyQ disorders are represented by 17 therapeutic approaches in the SCA group (together with DRPLA) and 12 therapeutic approaches for SBMA. The small number of therapeutic approaches for SCA can be explained by the low prevalence of all forms of hereditary SCA, which is estimated to be four cases per 100,000, whereas HD alone could have twice as many cases [322, 323]. Moreover, the absence of therapeutic investigations cannot simply be explained by the lack of suitable mouse models because there is a relatively broad range of choices. The models that exhibit a relatively rapid and broad phenotype include the B05 [324], SCA1 154/2 [325], SCA7 266Q/5Q [326], DRPLA Atro-118Q [271], and Q129 [327] models. However, with the exceptions of the B05 and SCA1 154/2 models, these models are not available in commercial repositories (Jackson Laboratories), which may partially explain why they are not as widely studied.

### Suitability of PolyQ Mouse Models for Experimental Therapies

There is no simple answer to the question of which model is the best suited for testing an experimental therapy. For example, would N171-82Q animals that have truncated gene, artificial promoter, and with a significantly reduced lifespan (a good therapeutic marker) be more useful than knock-in mice that are etiologically more similar to human HD but that exhibit a mild and relatively slow phenotype? Do the hyper-/hypobiphasic activity and marked cognitive deficits displayed by YAC128 mice qualify these animals as an ideal therapeutic model and eliminate the other models with phenotypes that are severe but less natural?

The concept of validity, adapted from Paul Willner’s work for use in the polyQ field, can be used to identify the strengths and weaknesses of a mouse model and help answer questions about its therapeutic suitability [328, 329], see also part I of this review). According to Willner, three main validation criteria can be used to compare and describe the usefulness of any given mouse model: construct validity, face validity, and predictive validity. Although the original idea and the definitions of different forms of validity may at first sound complicated, the concept is quite intuitive [4, 330].

The construct validity of genetically engineered models may be understood as the degree of etiological similarity between polyQ animals and the human condition. The major feature of the polyQ disease etiology is the underlying mutation represented by CAG repeats encoding the glutamine stretches in polyQ models. Almost all models express an elongated polyglutamine stretch in various genes, but the variable length of this stretch also contributes to variable degrees of construct validities with respect to different disorders. However, there are several other differences between models contributing to the degree of construct validity. For example, the construct validity of the N171-82Q model is reduced because it utilizes a nonnative promoter that drives the expression of truncated protein, whereas the expression of full-length human huntingtin driven by native promoter and regulatory sequences in YAC128 animals gives them a higher score in terms of construct validity. Obviously, criteria for such validation are rather subjective (see part I of this review). How, then, does the construct validity impact the therapeutic suitability of polyQ models? First, it affects the severity of the phenotype in mice. Truncated models seem to produce aggressive phenotypes, some aspects of which may not be induced by molecular mechanisms that are related to a particular disease but may instead be the effect of general polyglutamine toxicity. Second, the lack of some gene or protein sequences precludes selected polyQ models from being useful for testing certain therapeutic strategies. For example, all R6 models or N171-82 animals cannot be used to test the in vivo efficacy of RNAi reagents targeting 3´ region of *HTT* mRNA or to test the use of intrabodies that are specific to the C-terminus of human huntingtin, simply because these mice do not have the sequence (target) of interest.

The face validity of mouse models is the degree of similarity between the disease phenotype observed in mice and the pathophysiology and symptomatology that occur in patients. Mice differ from humans in their genetic, physiological, and anatomical features, which may significantly influence the disease presentation. The use of mice to mirror human disorders has inherent inaccuracies and is only a rough approximation of naturally existing conditions. The open question is whether the significant differences that are observed between models with high and low degree of face validity can be considered therapeutically or biologically relevant in light of a much greater distance between model animals and patients. Nevertheless, it seems that models that are genetically closer to humans (models that possess higher construct validity) also possess a more accurate phenotype (higher face validity), which is rather slow and mild. Unfortunately, using such animals slows down the data collection and publication processes and is one of the reasons why the vast majority of therapeutic approaches are tested using animals that display severe phenotypes that are manifested early, such as the R6/2, R6/1, and N171-82Q mice (Fig. 8). Furthermore, both the severity of phenotypes and the time at which they occur during the course of disease are related to the disease penetrance, and consequently, they are related to the degree of variation between individual animals. A slow disease progression raises the possibility that small phenotypic differences caused by external sources (e.g., housing conditions) accumulate and eventually amplify undesirable phenotypic variability. In contrast, the reduced lifespan observed in “fast models” may narrow the breeding window to a period of as short as 3–4 weeks in R6/2 males. In addition, severe phenotypes, which weaken the animals’ overall health, may lead to infertility and problems with gestation. Thus, these models tend to be more problematic in terms of breeding and maintenance (Tables 7 and 8). Overall, the suitability of polyQ mouse models for testing a preclinical therapy with respect to their face validity is not straightforward; sometimes, strong markers of the therapeutic outcome (such as an extremely shortened lifespan) are desirable even at the expense of their face validity.

**Table 7:** Summarizes the suitability of the mouse models of HD for the evaluation of experimental therapies

	N171-82Q	R6/1	R6/2	YAC128	YAC72	CAG140	Q111	BACHD	HD190QG
Construct validity—genetic similarity to the human patients (full-length protein/natural promoter/targeted transgene integration)	−/−/−	−/+/−	−/+/−	+^h^/+/−	+^h^/+/−	+^hb^/+/+	+^hb^/+/+	+^h^/+/−	−/+/−
Face validity—phenotypic similarity to the human patients (specific cell loss/rotarod impairment/ cognitive alterations)	+/+/−	−/+/+	−/+/+	+/+/+	−/+/−	+/+/+	−/+/−	−/+/−	−/−/−
Number of therapeutic approaches published	42	30	139	21	1	1	1	5	2
Number of phenotypes identified	64	71	170	51	12	44	15	23	11
Phenotype progression (AD50; age at 50 % detected phenotypes)	13	16	8	35	41	30	66	17	8
Breeding and husbandry (severe phenotype/reduced fertility)	+/nr	nr	+/+	nr	nr	nr	nr	nr	+/nr

This suitability can be evaluated by using construct validity (genetic similarities), face validity (phenotypic similarities), and predictive validity (cannot be determined at present). Moreover, this suitability can be assessed by the number of therapeutic approaches published (based on the data table) and the number of phenotypes identified (based on the data table in part I of the review). Additionally, the AD50 parameter (expressed as the number of weeks) reflects the disease dynamic in the models (see review part I for detailed information). The separate issue in assessing the therapeutic suitability in mouse models is the model maintenance and breeding

*h* human sequence, *hb* hybrid human/mouse sequence, *nr* not reported, *ret* 90Q R7E phenotype is limited to the eye retina

**Table 8:** Summarizes the suitability of other polyQ diseases for the evaluation of experimental therapies

	SCA1 B05	SCA1 154Q/2Q	SCA2 58Q	SCA3 polyQ69	SCA3 MJD84.2	SCA3 70.61 CAG	SCA3 Q79	SCA7 90Q R7E	SCA17 L7-hTBP	SBMA AR-97Q	SBMA 112Q	DRPLA Atro 118Q
Construct validity—genetic similarity to the human patients (full length protein/natural promoter/targeted transgene integration)	+^h^/−/−	+^hb^/+/+	+^h^/−/−	−/−/−	+^h^/+/−	+^h^/−/−	+^h^/−/−	+^h^/−/−	+/−/−	+^h^/−/−	+/−/−	+^h^/−/−
Face validity—phenotypic similarity to the human patients (specific cell loss/rotarod impairment/ cognitive alterations)	+/+/+	+/+/+	+/+/−	−/+/−	+/+/−	−/+/−	−/+/−	−/−/− (ret)	+/+/−	−/+/−	+/+/+	−/+/−
Number of therapeutic approaches published	6	8	2	1	1	1	1	2	1	11	1	1
Number of phenotypes identified	18	27	10	12	36	18	17	14	24	25	23	19
Phenotype progression (AD50; age at 50 % detected phenotypes)	10	19.5	25	3	41	13	30	3.25	13	10.5	30	11.5
Breeding and husbandry (severe phenotype/reduced fertility)	+/nr	+/nr	nr	+/nr	nr	+/nr	nr	nr	+/nr	+/nr	−/+	+/nr

This suitability can be evaluated by using construct validity (genetic similarities), face validity (phenotypic similarities), and predictive validity (cannot be determined at present). Moreover, this suitability can be assessed by the number of therapeutic approaches published (based on the data table) and the number of phenotypes identified (based on the data table in part I of the review). Additionally, the AD50 parameter (expressed as the number of weeks) reflects the disease dynamic in the models (see review part I for detailed information). The separate issue in assessing the therapeutic suitability in mouse models is the model maintenance and breeding

*h* human sequence, *hb* hybrid human/mouse sequence, *nr* not reported, *ret* 90Q R7E phenotype is limited to the eye retina

If we assume that instead of assessing the absolute face validity of a given model we can evaluate the validity of certain aspects of its phenotype, then the important question becomes one of determining which model best reflects the particular phenotypes that can be used as the indicators of the therapy outcome. For phenotypes that were the most often analyzed in therapeutic approaches using mouse models of polyQ disease see Fig. S1. Table 9 shows the frequencies of the individual phenotypes that were analyzed in the four HD mouse models that are most extensively used in studies of polyQ therapy: R6/2, N171-82Q, R6/1, and YAC128. How frequently a certain phenotype is tested tells about its detectability in the model and its usefulness as a potent marker of a therapeutic intervention. Rotarod analyses are commonly conducted in all four models revealing that the balance and coordination impairment are strongly reflected in all of these animals. Rotarod performance is used as a phenotypic improvement marker in studies of 66–79 % of all therapeutic approaches.

**Table 9:** Phenotypes commonly used as therapeutic outcome indicators

	N171-82Q	R6/1	R6/2	YAC 128
Brain atrophy	39 %	22 %	39 %	**63 %**
Cell loss	20 %	7 %	4 %	**58 %**
PolyQ aggregates	48 %	40 %	**54 %**	26 %
Brain weight	5 %	30 %	17 %	37 %
Rotarod test	**78 %**	**66 %**	**69 %**	**79 %**
Stride abnormalities	22 %	18 %	20 %	32 %
Locomotor impairment	10 %	19 %	32 %	26 %
Grip strength	7 %	4 %	13 %	5 %
Clasping	10 %	40 %	24 %	16 %
Learning deficits	2 %	15 %	13 %	26 %
Premature death	**85 %**	4 %	**70 %**	0 %
Body weight loss	**71 %**	**63 %**	**76 %**	12 %

Table 9 demonstrates how frequently a certain phenotype is tested to reveal the therapeutic outcome. This indirectly indicates the detectability and usefulness of a given phenotype in mice as a potent marker of a therapeutic intervention. Note that when a particular phenotype was tested in an individual therapeutic approach several times (e.g., using different methods), it was counted in the table only once. Phenotypes that were frequently selected as therapeutic intervention markers (in more than 50 % of the approaches) are marked in bold

Although an interpretation of the frequency with which various phenotypes are studied in YAC128 mice may be imprecise because of the small number of therapeutic approaches (only 21), some general trends may be observed. In contrast to R6/2 and N171-82 mice, the YAC128 model does not allow us to interpret therapy effectiveness by studying the survival rate or body weight losses. On the other hand, the YAC128 model is more suitable for testing the impact of the evaluated therapeutics on learning deficits and neuronal cell loss. Interestingly, the neuronal cell loss is used as an indicator of therapeutic effectiveness only in seven out of 120 therapeutic approaches in R6/2 animals that were collected in the data table, which indirectly indicates a lack of cell loss in R6/2 model (Table 9).

When discussing the suitability of mouse models for preclinical therapy, the most relevant among Willner’s criteria is probably that of predictive validity. This form of validity can be used to describe how well a test predicts future performance. For the purpose of analyzing the therapeutic usefulness of a mouse model, this question can be changed to: is the model capable of predicting the efficacy of a therapeutic intervention in human trials? Answering this question is not trivial; to validate a mouse model according to its predictive abilities, it is necessary to compare the outcomes of therapeutic interventions between the model and humans. Unfortunately, whereas numerous preclinical trials have been conducted in polyQ mouse models, only a few clinical trials have been conducted in patients [331]. Moreover, because there may be differences between mice and humans in both the optimal doses and pharmacokinetics of various therapeutic agents, the comparison and interpretation of study results may be difficult. Nevertheless, our data table, which lists the therapeutic outcomes of over 250 preclinical trials, may be used as a basis for the assessment of the predictive validities of polyQ mouse models as soon as more information about human trials is published.

## Conclusions

The past two decades of intensive study of polyQ diseases have revealed the genetic background of these diseases, uncovered many aspects of their pathogenesis, and have brought forth a broad spectrum of animal models. Unfortunately, an effective cure is still not available, and several intriguing questions remain unsolved. What we know is that expanded polyglutamine proteins alter many different cellular processes, which has important therapeutic implications. The pathomechanisms involved in disease development and progression are complex, independent, and often occur in parallel. Examples of such mechanisms are transcriptional deregulation, clearance machinery deficiency, and mitochondrial impairment. Therefore, a successful therapeutic approach should probably target multiple aspects of disease pathogenesis. Introducing genetic mouse models into the polyQ field has facilitated understanding of the etiologies of polyQ diseases and has accelerated the design and testing of new therapeutic approaches. In this work, we have reviewed approximately 250 therapeutic interventions that have been studied in mouse models of polyQ diseases. Our review is supported by the data table that contains over 2,000 records describing the in vivo therapeutic approaches. The data table includes detailed information about the mouse models, therapeutic strategies, methods of testing, outcomes, phenotypes used to test the outcomes, active substances, and their targets. Although the vast majority of therapeutic approaches have involved mouse models of HD, there are some common therapeutic approaches that were tested in other diseases. We believe that integrating the information about polyQ therapy in one framework can provide a new perspective for therapeutic research.

Therapeutic references not directly cited in text, [332–391].

## Electronic supplementary material

Below is the link to the electronic supplementary material.

ESM 1 (DOCX 21 kb)

ESM 2 (DOCX 23 kb)

ESM 3 (DOCX 20 kb)

ESM 4 (DOCX 23 kb)

ESM 5 (DOCX 23 kb)

ESM 6 (DOCX 20 kb)

ESM 7 (DOCX 21 kb)

ESM 8  (DOCX 25 kb)

ESM 9 (DOCX 23 kb)

ESM 10 (XLSX 299 kb)

Fig. S1 The graph demonstrates the phenotypes in mice that were used as a measuring the outcomes of polyQ therapies. Note that the R6/1, R6/2, and N171/82Q HD models were used in 80 % of all tests of polyQ treatment approaches; therefore, the analyzed phenotypes and their testing frequencies mirror the phenotypes that occurred in these mice (JPEG 45 kb)

High resolution image (TIFF 6.40 MB)
