# Supplementary material for: Mouse Models of Polyglutamine Diseases in Therapeutic Approaches: Review and Data Table. Part II
Source: Mol Neurobiol. 2012 Sep 4;46(2):430–66. doi: 10.1007/s12035-012-8316-3 (PMC3461214; doi:10.1007/s12035-012-8316-3)
Supplement: Supplementary file 1 — (DOCX 21 kb) [file 12035_2012_8316_MOESM1_ESM.docx]

**Supplementary information**

**Table design**

To create the data table of therapeutic approaches conducted on mouse models of polyQ diseases we have selected Excel format, because it is intuitive and well accessible to broad scientific audience that mostly need no introduction to immediately start work with such data table. We concluded that advantage of accessibility, ease of use and the possibility of self-modification prevail over the obvious drawbacks of Excel based lists.

For this data table we have analyzed ~180 research papers that report experimental therapy conducted on 21 models of polyQ diseases. In the present review, a therapeutic approach is understood as a pharmacological or non-pharmacological intervention performed on the individual mouse model. For example, if particular therapeutic paper tests one drug on two models, it is counted as two therapeutic approaches and appears in the data table twice. Analogically, when one paper presents separate evaluations of two different drugs on one mouse model, it also appears on the table as two different therapeutic approaches.

Our therapeutic data table focuses on disease phenotypes that are used as readouts of the therapy effectiveness. Each phenotype is assigned into one of the four main categories: motor, neuropathology, cognitive or other, which, for better clarity, are depicted in different colors. Additionally, every phenotype is characterized by the column “Methods” that presents method used to collect relevant data such as „rotarod” or „open field test”, as well as the column “Quantified parameter” that more accurately describes what exactly is quantified in order to characterize each phenotype (”latency to fall (s)” or „total distance moved (cm)”). The outcome of an experimental therapy is shown in columns “MUT vs. WT” and “TREATEMENT vs. MOCK”. The first of them displays the trend of the parameter change in transgenic mice compared to wild type animals and, in other words, shows how severe is the disease in the particular mouse model. Second column displays the trend of the parameter change in transgenic mice treated with the therapeutic drug compared to the mock-treated transgenic animals, therefore shows how effective the therapy is. To avoid any uncertainty, additional “Therapeutic outcome” column indicates whether the testing approach results in amelioration (beneficial, B) or deterioration (deleterious, DL) of analyzed phenotype or does not have any significant impact at all (not beneficial, NB). Next columns indicate what drugs (or approach) is used in each study and describe their detailed characteristic (i.e. drug feature and the route of administration). Column “Therapeutic strategy” groups all therapeutic approaches into categories in respect to the particular pathway responsible for the pathogenesis that they target.

Our data table is not the databases but when necessary it can be converted to SQL-type database files by the end user. Moreover our data table possesses many features of a database and the sets of defined information can be easily retrieved using either filter functions or pivot tables and pivot charts.

**The methods of retrieving the information from the multicolumn table**

The data table is constructed in Excel and therefore uses its common built-in filter functions for the most basic data retrieval and comparison. The filters are accessible from the first row of the excel sheet allowing the selection of the criteria from drop-down lists or applying sorting. The first “number” column holds the record numbers that allow for restoring the initial order of records. The data table can be filtered and sorted to retrieve the data of interest. For example, by applying filter on the “Therapeutic strategy” column, one can select specific strategy and get the overview of all therapeutic papers evaluating it, including such information as drugs and mouse models used in evaluating a strategy and general outcome of therapeutic intervention.

To perform more complicated data search and comparison we recommend using pivot tables and pivot charts that are one of the most powerful tools in Excel. The pivot tables were made to use with the numeric data but it can also be used for grouping and integrating text data from multiple columns of spreadsheet into one column in pivot table that creates tree-like structure allowing for uncovering the relation between the data. The easiest example is integrating two spreadsheet columns such as “disease” and “mouse model”. After using the function “insert pivot table” and selecting columns to be integrated the pivot table will display list of mouse models for every disease used in experimental therapies (see Sheet2 of the data table). Additionally it is possible to apply filter and for example display only HD and SCA3 models. Above example integrates only 2 columns but it is possible to construct research strategies integrating 4 or more columns into the one pivot table column. Such strategy combined with filtering can uncover quite complicated relation of data and is similar to querying the database. The relations and datasets can subsequently be retrieved to the separate table or spreadsheet for further processing. The pivot table can also be used for all operations on the data table where counting of records is important and to do this it is sufficient to drag the required column into the numeric field of pivot table. This strategy combined with the tree-like structure of text data and filters allows for finding numeric relation in text data. To illustrate the data retrieval from pivot table we have created a pivot table located in a separate sheet in our data table file.

**The phenotypes suitable for therapeutic approaches in polyQ models**

It is essential to possess a good understanding of the mouse model and the disease phenotypes to reveal and properly interpret the therapeutic outcome. The detailed characterization of polyQ mouse models and their phenotypes is already provided in our PART I data table and review. There we used an intensive system consisting of 6 columns to describe a single phenotype, which included the phenotype details and the locations of the phenotype in the CNS and non-CNS tissues. For the present therapeutic data table, we have simplified the phenotype description to focus on the therapeutic strategy, active substances and the therapy outcome. Therefore, the abnormalities tested in various *in vivo* therapeutic approaches are now described by 3 columns labeled “general phenotype”, “phenotype” and the “methods” (that were used to test the phenotype), omitting the “detailed phenotype” and the locations of the phenotype because these data were presented in the PART I table and review. For successful therapy, it is also essential to determine which of the phenotypes existing in transgenic animals need to be selected to reveal the outcome of the therapy and, second, how to test the phenotypes that are “the best choice” for the therapy. Therefore, we have analyzed the phenotypes and revealed the most frequently tested phenotypes and the testing methods used in the listed polyQ therapeutic studies.

One class of phenotypes that is present in patients of polyQ disorders can be summarized as “neuropathology”. These phenotypes are represented in mouse models, and the most tested phenotype is the group of neuronal characteristics that we have called the “neuronal physiology alterations”. This group of phenotypes contains mainly the molecular characteristics of neurons, such as the quantification of mRNAs and proteins that influence neuronal physiology, and is examined as the outcome of the experimental therapy. The most frequently examined molecules are polyQ proteins; histones and their acetylated forms; membrane receptors, such as dopamine receptors; growth factors, such as BDNF; chaperones, such as Hsp70 and 90; striatal marker DARPP-32; proteins in important transduction pathways, such as CREB, Akt, and Rho; and protein phosphorylation. However, the examination of the therapy outcome in this group depends strongly on the individual therapeutic strategy analyzed. The second phenotype that is often examined and is more universal as a therapy indicator is “brain atrophy”, assessed by stereological measurement, weight measurement, histochemistry and MRI. In this group of phenotypes, whole-brain atrophy or selective atrophy of various brain regions is measured by assessing the volume, area or thickness of cell layers. The brain regions that are examined most frequently include the striatum, cerebral cortex, cerebellum and brain ventricles. The third neuropathological phenotype that is examined most commonly is “polyQ protein aggregates”, assessed by immunohistochemistry and stereological measurement. Our data table contains 144 records that examined “polyQ protein aggregates” after the application of therapeutic substances. Of these, 78 records showed a decrease in aggregates after applying the treatment, 60 showed no difference, and 6 showed an increase in “polyQ protein aggregates”. Regardless of the role of aggregates in pathogenesis, this indicates that potentially beneficial therapeutic strategies may be associated with decreases in pQ protein aggregates or the reversal of already formed aggregates.

Another class of phenotypes that is also present in patients and mouse models are “motor” abnormalities. The most frequently tested phenotypes in this group are “balance and coordination alterations”, which are tested using the rotarod and beam walk test. The rotarod is a sensitive test for examining the motor function of the animals. Our data table I shows that motor impairment occurs in many mouse models even before the animals show clearly visible abnormalities. Moreover, the rotarod test is easy to perform and can be simply quantified. The second most commonly tested phenotype in this class is “locomotor impairment”, assessed by the open field test and activity chambers. Many parameters are recorded, including behavior counting, such as rearing, climbing, and grooming; or the locomotor distances are measured, such as the distance traveled, number of times crossing a certain point and velocity. In addition, the clasping behavior and gait alteration are tested by the tail suspension test and footprint test, respectively. Additionally, the “grip strength impairment” is tested by the grip strength meter or hanging wire test.

On average, the “cognitive” group of phenotypes is not frequently tested. The most common phenotypes are spatial learning deficits (42 records), which are tested by the Morris water maze, Y-maze task and T-maze alternation task; exploratory behavior impairment (22 records), tested by the open field test and elevated maze; and affective functional alteration (6 records), tested by the Porsolt forced swim test. The reason for the rare use of these tests in therapeutic approaches is because these tests are not easy to standardize and set up.

The most frequently tested phenotypes from the category “other” include body weight loss (158 records), premature death (138 records), muscle abnormalities (53 records) and diabetes mellitus (37 records).

| **Columns** | **Description** |
| --- | --- |
| **Number** | The record number |
| **Disease** | The disease that is modeled by the mouse model |
| **Mouse model** | The mouse model that was used for experimental therapy |
| **General phenotype** | The phenotyoes in this column are divided in four categories: motor, neuropathology, cognitive and other |
| **Phenotypes** | This column lists detailed phenotypes that were tested to assess the outcome of the experimental therapy. The phenotypes fall into the 4 categories of general phenotypes |
| **Methods** | The methods that were used to test the detailed phenotype |
| **Quantified parameter** | The exact parameter that was quantified to assess the outcome of the therapy |
| **MUT vs. WT** | This column records whether the tested detailed phenotype was diminished or strengthened in mutant animals |
| **TREATEMENT vs. MOCK** | This column records the outcome of the therapy |
| **Therapeutic outcome** | This column specify the outcome of the therapy |
| **Drug/aim** | The active substance used in experimental therapy |
| **Drug feature** | Information on the direct drug target or other drug features |
| **Therapeutic strategy** | The general therapeutic strategy |
| **Delivery route** | The way the active substance was administrated to transgenic animals |
| **Reference** | The reference to the original work describing the therapeutic approach |
| **Year** | The year of the referenced work |

Supp. Table 1. Descriptions of the 15 columns present in the Excel-based data table.
