# Supplementary material for: Mouse Models of Polyglutamine Diseases in Therapeutic Approaches: Review and Data Table. Part II
Source: Mol Neurobiol. 2012 Sep 4;46(2):430–66. doi: 10.1007/s12035-012-8316-3 (PMC3461214; doi:10.1007/s12035-012-8316-3)
Supplement: Supplementary file 2 — (DOCX 23 kb) [file 12035_2012_8316_MOESM2_ESM.docx]

| Supplementary table 2. Drugs used in clearance machinery-related approaches | | | | |
| --- | --- | --- | --- | --- |
|  | Drug | Drug target/feature | Mouse model | Reference |
| Chaperone related polyQ protein turnover | Hsp70 and/or HDJ2 overexpression | Heat shock protein | 90Q R7E | Helmlinger et al. 2004 |
|  | Hsp70 overexpression | Heat shock protein | B05 | Cummings et al. 2001 |
|  | Hsp70 overexpression | Heat shock protein | R6/2 | Hansson et al. 2003; Hay et al. 2004 |
|  | Hsp104 overexpression | Heat shock protein | N171-82Q | Vacher et al. 2005 |
|  | 17-AAG | Hsp90 inhibitor | AR-97Q | Waza et al. 2005 |
|  | 17-DMAG | Hsp90 inhibitor | AR-97Q | Tokui et al. 2009 |
|  | GGA | HSPs expression inducer | AR-97Q | Katsuno et al. 2005 |
|  | BAG1 | Hsp70 regulator | N171-82Q | Orr et al. 2008 |
|  | HSF1 overexpression | heat shock transcription factor | R6/2 | Fujimoto et al. 2005 |
|  | DNAJB | neuronal chaperone HSJ1a | R6/2 | Labbadia et al. 2012 |
| Inhibition of polyQ protein aggregation | Chlorpromazine | D2 receptor antagonist, prion inhibitor | N171-82Q | Schilling et al. 2004 |
|  | C2-8 | polyQ aggregation inhibitor | R6/2 | Chopra et al. 2007 |
|  | PGL-135 | polyQ aggregation inhibitor | R6/2 | Hockly et al. 2006 |
|  | Riluzole | Voltage-gated sodium channels stabilizer | R6/2 | Hockly et al. 2006 |
|  | Trehalose | aggregation-preventing disaccharide | R6/2 | Yang and Yu 2009, Tanaka et al. 2004 |
|  | Minocycline and Doxycycline | Caspase-1 inhibitors, anti-amyloid compound | R6/2 | Smith et al. 2003 |
|  | Congo red | anti-amyloid compound | R6/2 | Sánchez et al. 2003; Wood et al. 2007 |
|  | Y-27632 | ROCK inhibitor | R6/2 | Li et al. 2009 |
|  | Antp-QBP1 | polyQ-binding peptide | R6/2 | Popiel et al. 2009 |
|  | Happ1 intrabody | recognition of polyP and P-rich Htt domains | N171-82Q; R6/2; YAC128; BACHD | Southwell et al. 2009 |
|  | VL12.3 intrabody | recognition of the N terminus of Htt | YAC128; R6/2 | Southwell et al. 2009 |
|  | scFv-EM48 intrabody | recognition of mutant Htt | N171-82Q | Wang et al. 2008 |
|  | scFv-C4 intrabody | recognition of the N terminus of Htt | R6/1 | Snyder-Keller et al. 2010 |
| polyQ protein turnover enhancement (autophagy) | Temsirolimus (CCI-779) | mTOR inhibitor | 70.61 CAG | Menzies et al.. 2010 |
|  | Temsirolimus (CCI-779) | mTOR inhibitor | N171-82Q | Ravikumar et al. 2004 |
|  | Rilmenidine | mTOR-independent autophagy inducer | N171-82Q | Rose et al. 2010 |
|  | QBP1-HSC70bm peptide | targets pQ protein for chaperone-mediated degradation | R6/2; HD190QG | Bauer et al. 2010 |
|  | Everolimus | mTOR inhibitor | R6/2 | Fox et al. 2010 |
| polyQ protein turnover enhancement (UPS) | CHIP overexpression | HSPs binding and pQ ubiquitination | AR-97Q | Adach et al. 2007 |
|  | IGF-1 | (PI3K)/Akt pathway activator | AR-97Q  R6/2 | Palazzolo et al. 2009  Duarte et al. 2011 |
|  | CRAG overexpression | Ubiquitin-proteasome system activator | polyQ69 | Torashima et al. 2008 |
|  | Benzamil (Ben) | highly specific ASIC blocker | R6/2 | Wong et al. 2008 |
|  | REGg depletion | Proteasomal cleavage inhibitor | R6/2 | Bett et al. 2006 |
