# Supplementary material for: Mouse Models of Polyglutamine Diseases in Therapeutic Approaches: Review and Data Table. Part II
Source: Mol Neurobiol. 2012 Sep 4;46(2):430–66. doi: 10.1007/s12035-012-8316-3 (PMC3461214; doi:10.1007/s12035-012-8316-3)
Supplement: Supplementary file 3 — (DOCX 20 kb) [file 12035_2012_8316_MOESM3_ESM.docx]

| Supplementary table 3. Drugs used in the downregulation of polyQ protein expression | | | |
| --- | --- | --- | --- |
| Drug | Drug target/feature | Mouse model | Reference |
| anti-huntingtin siRNA | RNA interference reagent | R6/2 | Wang et al. 2005 |
| anti-huntingtin shRNA | RNA interference reagent | R6/1 | Rodriguez-Lebron et al. 2005 |
| anti-huntingtin mi-shRNA | RNA interference reagent | N171-82Q | Boudreau et al. 2009 |
| anti-huntingtin shRNA | RNA interference reagent | N171-82Q | Harper et al. 2005 |
| anti-ataxin-1 shRNA | RNA interference reagent | B05 | Xia et al. 2004 |
| Anti-EGFP shRNA | RNA interference reagent | HD190QG | Machida et al. 2006 |
| Clioquinol | metal-binding compound, CLK-1 inhibitor | R6/2 | Nguyen et al. 2005 |
| anti-huntingtin SNP-ASO | Antisense oligonucleotide | BACHD | Carroll et al. 2011 |
| anti-huntingtin ASO | Antisense oligonucleotide | YAC128, BACHD, R6/2 | Kordasiewicz et al. 2012 |
