# Supplementary material for: Mouse Models of Polyglutamine Diseases in Therapeutic Approaches: Review and Data Table. Part II
Source: Mol Neurobiol. 2012 Sep 4;46(2):430–66. doi: 10.1007/s12035-012-8316-3 (PMC3461214; doi:10.1007/s12035-012-8316-3)
Supplement: Supplementary file 4 — (DOCX 23 kb) [file 12035_2012_8316_MOESM4_ESM.docx]

| Supplementary table 4. Drugs used in energy metabolism and free radicals-related approaches | | | | |
| --- | --- | --- | --- | --- |
|  | Drug | Drug target/feature | Mouse model | Reference |
| Anti-oxidation | nNOS depletion or knockout | Catalyzes nitric oxide synthesis from L-Arg | R6/1 | Deckel et al. 2002 |
|  | S-methylisothiourea (SMT) | iNOS inhibitor | R6/1 | Deckel et al. 2002 |
|  | Ascorbate (Vitamin C) | Free-radical scavenger | R6/2 | Rebec et al. 2003 |
|  | Arginine | NO level modulator | R6/1 | Deckel et al. 2000 |
|  | dimethylfumarate | inducer of phase 2 detoxifying enzymes | R6/2; YAC128 | Ellrichmann et al. 2011 |
|  | Nordihydroguaiaretic acid (NDGA) | antioxidant compound | R6/2 | Lee et al. 2011 |
|  | tetrathiomolybdate | copper complexing agent | R6/2 | Tallaksen-Greene et al. 2009 |
| Mitochondrial dysfunction/Anti-oxidation | Coenzyme Q10 | Free-radical scavenger | R6/2;  N171-82Q | Menalled et al. 2010; Smith et al. 2006; Schilling et al. 2004 |
|  | SOD1 overexpression | Free-radical scavenger | N171-82Q | Schilling et al. 2004 |
|  | Hsp27 overexpression | suppressesor of ROS formation | R6/2 | Zourlidou et al. 2007 |
|  | BN82451 | antioxidant, anti-inflammator | R6/2 | Klivenyi et al. 2003 |
|  | S-PBN | Free-radical scavenger | R6/2 | Andreassen et al. 2001 |
|  | L-Carnitine | Free-radical scavenger | N171-82Q | Vamos et al. 2010 |
|  | TUDCA | Antioxidant, Bax translocation inhibitor | R6/2 | Keene et al. 2002 |
|  | CDDO-EA and CDDO-TFEA | Nrf2/ARE antioxidant pathway inducer | N171-82Q | Stack et al. 2010 |
| Mitochondrial dysfunction | Gabapentin-lactam | Mitochondrial KATP agonist | R6/2 | Zucker et al. 2004 |
|  | CypD depletion | regulator of mitochondrial Ca2+-loading capacity | R6/2 | Perry et al. 2010 |
|  | Nortriptyline | mitochondrial permeability transition (mPT) inhibitor | R6/2 | Wang et al. 2007 |
|  | Irs2 depletion | Insulin/IGF signaling cascade componenet | R6/2 | Sadagurski et al. 2011 |
| Mitochondrial dysfunction/ energy metabolism | Creatine | Energy suplier | N171-82Q; R6/2 | Andreassen et al. 2001; Dedeoglu et al. 2003; Ferrante et al. 2000 |
|  | Dichloroacetate | PDC kinase inhibitor | R6/2;  N171-82Q | Andreassen et al. 2001 |
|  | Lipoic acid | Free-radical scavenger, Pyruvate and a-ketoglutarate dehygrogenaze co-factor | R6/2; N171-82Q | Andreassen et al. 2001 |
|  | Dietary restriction | Intermittent fasting | N171-82Q | Duan et al. 2003 |
|  | Optimization of Feeding Regimes | enhanced diet | R6/2 | Carter et al. 2000 |
|  | thiazolidinedione (TZD) | PPARgamma agonist | R6/2 | Chiang et al. 2010 |
|  | PN401 | Uridine prodrug | R6/2;  N171-82Q | Saydoff et al. 2006 |
|  | SRT501-M | SIRT1 activator | N171-82Q | Ho et al. 2010 |
|  | Nicotinamide (NAM) | SIRT1 inhibitor | R6/2 | Hathorn et al. 2011 |
|  | ketogenic diet (KD) | cellular energy level enhancer | R6/2 | Ruskin et al. 2011 |
|  | PGC-1a | transcriptional coactivator | NLS-N171-82Q | Chaturvedi et al. 2009 |
|  | Sirt1 | Transcriptional regulator | N171-82Q; BACHD | Jeong et al. 2012  Jiang et al. 2012 |
|  | D-b-Hydroxybutyrate | Mitochondrial energizing ketone body | R6/2 | Lim et al. 2011 |
