# Supplementary material for: Mouse Models of Polyglutamine Diseases in Therapeutic Approaches: Review and Data Table. Part II
Source: Mol Neurobiol. 2012 Sep 4;46(2):430–66. doi: 10.1007/s12035-012-8316-3 (PMC3461214; doi:10.1007/s12035-012-8316-3)
Supplement: Supplementary file 5 — (DOCX 23 kb) [file 12035_2012_8316_MOESM5_ESM.docx]

| Supplementary table 5. Drugs used in excitotoxicity and aberrant neurotransmission-related approaches | | | | |
| --- | --- | --- | --- | --- |
|  | Drug | Drug target/feature | Mouse model | Reference |
| Protection from excitotoxicity | ceftriaxone | cephalosporin antibiotic, GLT1 expression-enhancing | R6/2 | Sari et al. 2010 |
|  | Remacemide | NMDAR antagonist | N171-82Q | Schilling et al. 2004 |
|  | CP101606;  hydroxypropyl-β-cyclodextrin; ifenprodil;  Perzinfotel; RO25,6981 | NR2B antagonist | R6/2 | Tallaksen-Greene et al. 2010 |
|  | 6-OHDA induced SN lesions | Chemical lesion | R6/2 | Stack et al. 2007 |
|  | Cerebellar aspiration lesions;  Cortical lesions | Surgical lesion | R6/2 | Stack et al. 2007 |
|  | Riluzole | Voltage-gated sodium channels stabilizer | R6/2 | Schiefer et al. 2002 |
|  | LY379269 | mGluR2 agonist | R6/2 | Schiefer et al. 2004 |
|  | MPEP | mGluR5 antagonist | R6/2 | Schiefer et al. 2004 |
|  | Probenecid | MRP1 and MRP2 inhibitor | N171-82Q | Vamos et al. 2009 |
|  | SCH58261 | adenosine A2A receptor antagonist | R6/2 | Domenici et al. 2007; Cipriani et al. 2008, Gianfriddo et al. 2004 |
|  | CGS21680 | adenosine A2A receptor agonist | R6/2 | Chou et al. 2005;  Chiang et al. 2009 |
| Ca2+: intracellular level modulation | Dantrolene | Ca2+ channel inhibitor | SCA2 58Q | Liu et al. 2009 |
|  | Dantrolene | Ca2+ channel inhibitor | MJD84.2 | Chen et al. 2008 |
|  | Dantrolene | Ca2+ channel inhibitor | YAC128 | Chen et al. 2011 |
|  | IC10 | C-terminal fragment of InsP3R1 | YAC128 | Tang et al. 2009 |
| Neurotransmitter activity regulation | Tacrine, moclobemide and creatine | AChE inhibitor, MAO inhibitor, energy suplier resp. | R6/2 | Morton et al. 2005 |
|  | Cplx2 depletion or knockout | synaptic vesicle exocytosis stimulator | R6/2 | Glynn et al. 2007 |
|  | L-DOPA | dopamine precursor | YAC128 | Tang et al. 2007 |
|  | L-DOPA and TBZ | VMAT-inhibitor | YAC128 | Tang et al. 2007 |
|  | TBZ | VMAT-inhibitor | YAC128 | Wang et al. 2010 |
|  | Tiagabine | GABA reuptake inhibitor | N171-82Q | Masuda et al. 2008 |
|  | ceftriaxone | Cephalosporin antibiotic, GLT1 expression-enhancing | R6/2 | Miller et al. 2008 |
|  | HU210 and THC | cannabinoid receptor agonists | R6/1 | Dowie et al. 2010 |
|  | URB597 | inhibitor of endocannabinoid metabolism (FAAH) | R6/1 | Dowie et al. 2010 |
| protection from excitotoxicity/ mitochondrial dysfunction/ Anti-oxidation, | Remacemide  (with Coenzyme Q10) | NMDAR antagonist  (with free-radical scavenger) | N171-82Q R6/2 | Schilling et al. 2001; Ferrante et al. 2002 |
