# Supplementary material for: Mouse Models of Polyglutamine Diseases in Therapeutic Approaches: Review and Data Table. Part II
Source: Mol Neurobiol. 2012 Sep 4;46(2):430–66. doi: 10.1007/s12035-012-8316-3 (PMC3461214; doi:10.1007/s12035-012-8316-3)
Supplement: Supplementary file 6 — (DOCX 20 kb) [file 12035_2012_8316_MOESM6_ESM.docx]

| Supplementary table 6. Drugs used in apoptosis-related approaches | | | |
| --- | --- | --- | --- |
| Drug | Drug target/feature | Mouse model | Reference |
| Minocycline | Caspase-1 inhibitor | R6/2;  N171-82Q | Chen et al. 2000;  Wang et al. 2003;  Mievis et al. 2007; Menalled et al. 2010 |
| Mutant caspase-1 protein (M17Z) | Caspase-1 pathways inhibitor | R6/2 | Ona et al. 1999 |
| zVAD-fmk | Caspase inhibitor | R6/2 | Ona et al. 1999 |
| Minocycline (with Coenzyme Q10) | Caspase-1 inhibitor (with free-radical scavenger) | R6/2 | Stack et al. 2006 |
| Ethyl-EPA | derivate of the omega-3 fatty acid | YAC128 | Van Raamsdonket al. 2005 |
| Methazolamide | Inhibitor of mitochondrial cytochrome c release | R6/2 | Wang et al. 2008 |
| Necrostatin-1 | RIP1 kinase inhibitor | R6/2 | Zhu et al. 2011 |
| A-438079;  Brilliant Blue G | P2X7 receptor antagonist | R6/1 | Díaz-Hernández et al. 2009 |
