# Supplementary material for: Mouse Models of Polyglutamine Diseases in Therapeutic Approaches: Review and Data Table. Part II
Source: Mol Neurobiol. 2012 Sep 4;46(2):430–66. doi: 10.1007/s12035-012-8316-3 (PMC3461214; doi:10.1007/s12035-012-8316-3)
Supplement: Supplementary file 7 — (DOCX 21 kb) [file 12035_2012_8316_MOESM7_ESM.docx]

| Supplementary table 7. Drugs used in transcriptional deregulation-related approaches | | | | |
| --- | --- | --- | --- | --- |
|  | Drug | Drug target/feature | Mouse model | Reference |
| Chromatin remodeling | Mithramycin | Histones/DNA binding antibiotic | R6/2 | Ferrante et al. 2004 |
|  | Chromomycin and/or Mithramycin | Histones/DNA binding antibiotic | R6/2; N171-82Q | Stack et al. 2007 |
|  | HDAC7 depletion | Histone deacetylase | R6/2 | Benn et al. 2009 |
|  | SAHA | HDAC inhibitor | R6/2 | Benn et al. 2009 |
|  | Sodium butyrate | HDAC inhibitor | ataxin-3-Q79 | Chou et al. 2011 |
|  | Sodium butyrate | HDAC inhibitor | R6/2 | Ferrante et al. 2003 |
|  | Sodium butyrate | HDAC inhibitor | Atro 118Q | Ying et al. 2006 |
|  | Sodium butyrate | HDAC inhibitor | AR-97Q | Minamiyam et al. 2004 |
|  | Phenylbutyrate | HDAC inhibitor | N171-82Q | Gardian et al. 2005 |
|  | SAHA | HDAC inhibitor | R6/2 | Hockly et al. 2003  Mielcarek et al. 2011 |
|  | HDACi 4b | HDAC inhibitor | R6/2 | Thomas et al. 2008 |
|  | Valproate | HDAC inhibitor, GABA transaminase inhibitor | N171-82Q | Zádori et al. 2009 |
| Transcription regulation | Lithium chloride | GSK3b inhibitor | R6/2 | Wood and Morton 2003 |
|  | Lithium carbonate | GSK3b inhibitor | Sca1 154Q/2Q | Watase et al. 2007 |
|  | NP03 | Low-dose lithium formulation | YAC128 | Pouladi et al. 2012 |
|  | Sp1 knock-out | Transcriptional activator (interacts with Htt) | N171-82Q; R6/2 | Qiu et al. 2006 |
| cAMP signaling restoration | TP-10 | PDE10A inhibitor | R6/2 | Giampà et al. 2010 |
|  | rolipram | phosphodiesterase type IV inhibitor | R6/2 | Giampà et al. 2009; DeMarch et al. 2008 |
