# Supplementary material for: Mouse Models of Polyglutamine Diseases in Therapeutic Approaches: Review and Data Table. Part II
Source: Mol Neurobiol. 2012 Sep 4;46(2):430–66. doi: 10.1007/s12035-012-8316-3 (PMC3461214; doi:10.1007/s12035-012-8316-3)
Supplement: Supplementary file 8 — (DOCX 25 kb) [file 12035_2012_8316_MOESM8_ESM.docx]

| Supplementary table 8. Drugs used in 4 neuroprotection/ neuromodulation approaches | | | | |
| --- | --- | --- | --- | --- |
|  | Drug | Drug target/feature | Mouse model | Reference |
| Neurotrophic factor-induced neuroprotection | BDNF overexpression | Neurotrophic factor | YAC128; R6/1 | Xie et al. 2010;  Gharami et al. 2008 |
|  | BDNF depletion | Neurotrophic factor | R6/1 | Pineda et al. 2005;  Canals et al. 2004 |
|  | BDNF - exogenous | Neurotrophic factor | R6/1 | Canals et al. 2004 |
|  | Cells expressing GDNF | Neurotrophic factor | N171-82Q | Ebert et al. 2010 |
|  | Cells expressing BDNF | Neurotrophic factor | YAC128 | Dey et al. 2010 |
|  | Cells expressing NGF | Neurotrophic factor | YAC128 | Dey et al. 2010 |
|  | Neurturin | Neurotrophic factor | N171-82Q | Ramaswamy et al. 2009 |
|  | GDNF | Neurotrophic factor | R6/2;  N171-82Q | Popovic et al. 2005;  McBride et al. 2006 |
|  | CNTF | Neurotrophic factor | YAC72; R6/1 | Zala et al. 2004; Denovan-Wright et al. 2008 |
|  | FGF-2 | Neurotrophic factor | R6/2 | Jin et al. 2005 |
|  | BDNF and Noggin overexpression | Neurotrophic factor and BMPs inhibitor | R6/2 | Cho et al. 2007 |
|  | KP545 | pyrimidine substitute | R6/2 | Dey et al. 2007 |
|  | Sertraline | serotonin reuptake inhibitor | N171-82Q | Duan et al. 2008;  Cheng et al. 2011 |
|  | Sertraline | serotonin reuptake inhibitor | R6/2 | Peng et al. 2008 |
|  | Paroxetine | serotonin reuptake inhibitor | N171-82Q | Duan et al. 2004 |
|  | fluoxetine or imipramine | serotonin reuptake inhibitor | YAC128 | Pouladi et al. 2009 |
|  | Fluoxetine | serotonin reuptake inhibitor | R6/1 | Grote et al. 2005 |
|  | Electroconvulsive shock | Inducer of BDNF production | N171-82Q | Mughal et al. 2011 |
|  | Ampakine CX929 | AMPA-type glutamate receptor positive modulator | R6/2; CAG140 | Simmons et al. 2011;  Simmons et al. 2009 |
|  | VEGF overexpression | Neurotrophic factor | Sca1 154Q/2Q | Cvetanovic et al. 2011 |
| Enviromental enrichment-induced neuroprotection | Environmental enrichment |  | R6/1 | van Dellen et al. 2000;  Glass et al. 2004;  Spires et al. 2004;  Lazic et al. 2006  Benn et al. 2010 |
|  | Environmental enrichment |  | R6/2 | Carter et al. 2000;  Hockly et al. 2002;  Wood et al. 2010;  Zajac et al. 2010 |
|  | Environmental enrichment |  | N171-82Q | Schilling et al. 2004 |
|  | Physical exercise |  | R6/1; R6/2 | Pang et al. 2006;  van Dellen et al. 2008  Kohl et al. 2007;  Zajac et al. 2010 |
|  | Wheel running, enviromental enrichement |  | R6/1 | van Dellen et al. 2008 |
|  | Behavioural testing |  | R6/2 | Hunt and Morton 2005 |
|  | Exercise |  | Sca1 154Q/2Q | Fryer et al. 2011 |
| Cell therapy | Neural Precursor Cells |  | B05 | Chintawar et al. 2009 |
|  | Multipotent Stromal Cells |  | N171-82Q | Snyder et al. 2010 |
|  | Adipose stem cells |  | R6/2 | Lee et al. 2009 |
|  | Striatal grafts |  | R6/2 | Dunnett et al. 1998 |
|  | ACC transplatation |  | R6/1 | van Dellen et al. 2001 |
|  | Bone marrow-derived cells |  | Sca1 154Q/2Q; YAC128; BACHD | Chen et al. 2011  Kwan et al. 2012 |
|  | Human Mesenchymal Stem Cells |  | R6/2; SCA2 58Q | Lin et al. 2011  Chang et al. 2011 |
| Neurogenesis promotion | Neuropeptide Y | neurotransmitter | R6/2 | Decressac et al. 2010 |
| neurogenesis promotion/ protection from excitotoxicity, | Asialoerythropoetin | EPO variant without erythropoietic effects | R6/2 | Gil et al. 2004 |
