# Supplementary material for: Mouse Models of Polyglutamine Diseases in Therapeutic Approaches: Review and Data Table. Part II
Source: Mol Neurobiol. 2012 Sep 4;46(2):430–66. doi: 10.1007/s12035-012-8316-3 (PMC3461214; doi:10.1007/s12035-012-8316-3)
Supplement: Supplementary file 9 — (DOCX 23 kb) [file 12035_2012_8316_MOESM9_ESM.docx]

| Supplementary table 9. Drugs used in other approaches | | | | |
| --- | --- | --- | --- | --- |
|  | Drug | Drug target/feature | Mouse model | Reference |
|  | DNA vaccination | Immunization against toxic pQ protein | R6/2 | Miller et al. 2007 |
|  | Atxn1l overexpression | Mutant Ataxin-1 protein competitor | Sca1 154Q/2Q | Bowman et al. 2007 |
|  | CEP-1347 | Inhibitor of mixed lineage kinase (MLK) | R6/2 | Conforti et al. 2008;  Apostol et al. 2008 |
|  | fisetin | Ras-ERK cascade activator | R6/2 | Maher et al. 2011 |
|  | anti-Ask1-antibody | Inhibitor of Htt nuclear translocation factor | R6/2 | Cho et al. 2009 |
|  | TRTK12 | S100B inhibitory peptide | B05 | Parminder et al. 2011 |
|  | G- CSF | colony-stimulating factor hormone | L7-hTBP | Chang et al. 2011 |
| Diabetes | Glibenclamide and/or rosiglitazone | KATP channel inhibitor/PPAR receptors agonist | R6/2 | Hunt and Morton 2005 |
|  | Exendin-4 | GLP-1 receptor agonist | N171-82Q | Martin et al. 2009 |
|  | Metformin | Anti-diabetes drug | R6/2 | Ma et al. 2007 |
| Transglutaminase activity | Cystamine | transglutaminase inhibitor | R6/2 | Karpuj et al. 2002; Dedeoglu et al. 2002; Wang et al. 2005;  Bailey and Johnson 2006 |
|  | TG2 knock-out | transglutaminase activity inhibition | R6/1 | Mastroberardino et al. 2002 |
|  | Cystamine | transglutaminase inhibitor | YAC128 | Van Raamsdonk et al. 2005 |
|  | Cystamine and cysteamine | transglutaminase inhibitors | Q111; R6/1 | Borrell-Pagès et al. 2006 |
|  | mithramycin and cystamine | Histones/DNA binding antibiotic and transglutaminase inhibitor | R6/2 | Ryu et al. 2006 |
|  | calmodulin (CaM) fragment | CaM-htt interaction inhibitor | R6/2 | Dai et al. 2009 |
|  | ZDON | peptide-based transglutaminase inhibitor | YAC128 | McConoughey et al. 2010 |
| Testosterone level and activity | Flutamide | Androgen receptor agonist | AR-97Q | Katsuno et al. 2003 |
|  | Testosterone reduction | Androgen receptor ligand | AR-97Q | Katsuno et al. 2002 |
|  | Leuprorelin acetate | luteinising hormone releasing hormone (LHRH) agonist | AR-97Q | Katsuno et al. 2003 |
|  | Castration |  | AR-97Q; 112Q | Katsuno 2002; Chevalier-Larsen et al. 2004 |
| Sleep/wake cycle regulation | Alprazolam | Benzodiazepine (GABA receptor complex enhancer) | R6/2 | Pallier et al. 2007;  Pallier and Morton 2009 |
|  | chloral hydrate | GABA receptor complex enhancer | R6/2 | Pallier et al. 2007 |
|  | Modafinil (or with Alprazolam) | wake-promoting agents | R6/2 | Pallier and Morton 2009 |
| Inflammatory pathways | Acetylsalicylate | COX-1 inhibitor | N171-82Q; R6/2 | Norflus et al. 2004 |
|  | Rofecoxib | COX-2 inhibitor | R6/2 | Norflus et al. 2004 |
|  | Celecoxib | COX-2 inhibitor | N171-82Q | Schilling et al. 2004 |
