# Supplementary figures and images for: Mouse Models of Polyglutamine Diseases in Therapeutic Approaches: Review and Data Table. Part II
Source: Mol Neurobiol. 2012 Sep 4;46(2):430–66. doi: 10.1007/s12035-012-8316-3 (PMC3461214; doi:10.1007/s12035-012-8316-3)

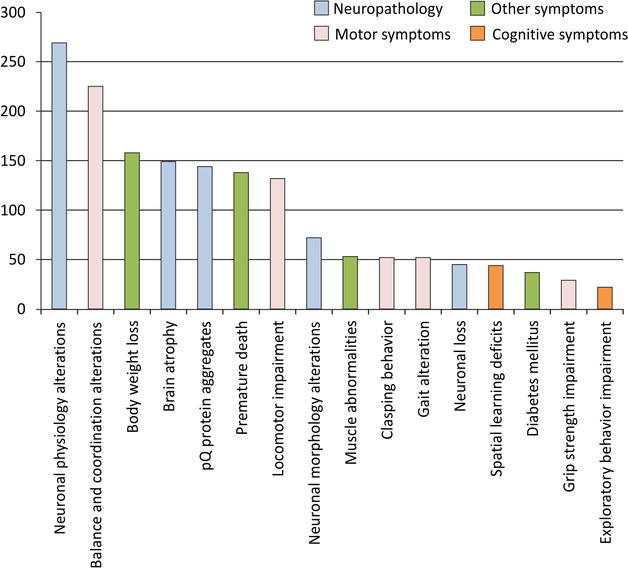

Supplement: Supplementary file 11 — The graph demonstrates the phenotypes in mice that were used as a measuring the outcomes of polyQ therapies. Note that the R6/1, R6/2, and N171/82Q HD models were used in 80 % of all tests of polyQ treatment approaches; therefore, the analyzed phenotypes and their testing frequencies mirror the phenotypes that occurred in these mice (JPEG 45 kb) [file 12035_2012_8316_Fig9_ESM.jpg]

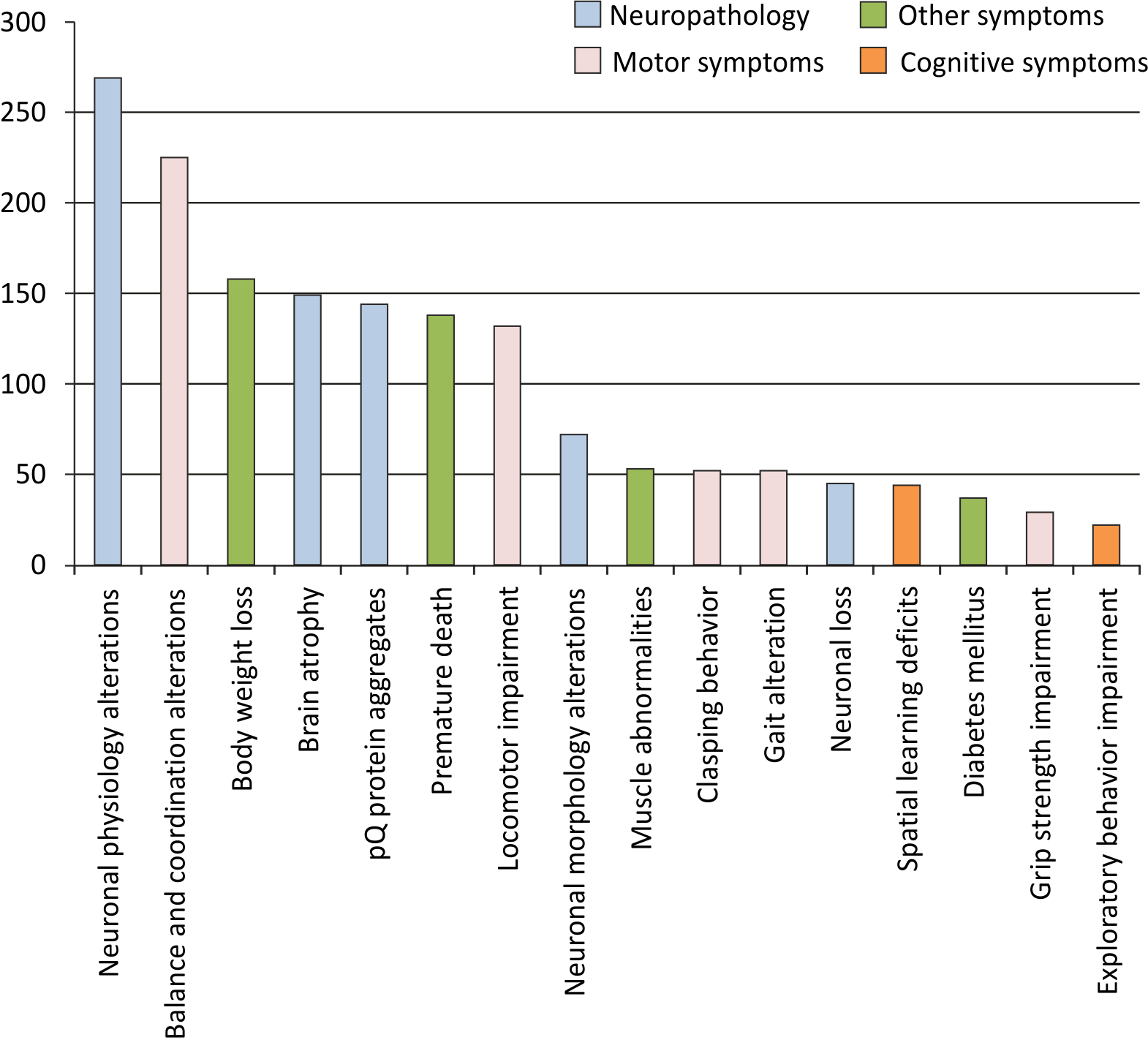

Supplement: Supplementary file 12 — High resolution image (TIFF 6.40 MB) [file 12035_2012_8316_MOESM11_ESM.tif]
